# Solvation Dynamics of Thermoresponsive Polymer Films: The Influence of Salt Series in Water and Mixed Water/Methanol Atmosphere

**DOI:** 10.1002/advs.202408073

**Published:** 2024-12-31

**Authors:** Peixi Wang, Tianle Zheng, Julija Reitenbach, Simon A. Wegener, Linus F. Huber, Lucas P. Kreuzer, Suzhe Liang, Robert Cubitt, Ya‐Jun Cheng, Tonghui Xu, Viet Hildebrand, André Laschewsky, Christine M. Papadakis, Peter Müller‐Buschbaum

**Affiliations:** ^1^ Chair for Functional Materials Department of Physics TUM School of Natural Sciences Technical University of Munich James‐Franck‐Str. 1 85748 Garching Germany; ^2^ Heinz Maier‐Leibnitz Zentrum (MLZ) Technical University of Munich Lichtenbergstr. 1 85748 Garching Germany; ^3^ Institut‐Laue‐Langevin 6 rue Jules Horowitz 38000 Grenoble France; ^4^ Ningbo Institute of Materials Technology & Engineering Chinese Academy of Sciences 1219 Zhongguan West Rd. Ningbo Zhejiang Province 315201 P.R. China; ^5^ Department of Chemistry College of Sciences. Shanghai University 200444 Shanghai P. R. China; ^6^ Institut für Chemie Universität Potsdam Karl‐Liebknecht‐Str. 24–25 14476 Potsdam‐Golm Germany; ^7^ Fraunhofer Institut für Angewandte Polymerforschung Geiselbergstr. 69 14476 Potsdam‐Golm Germany; ^8^ Soft Matter Physics GroupDepartment of Physics TUM School of Natural Sciences Technical University of Munich James‐Franck‐Str. 1 85748 Garching Germany

**Keywords:** Hofmeister series, hydration shell, neutron reflectivity, potassium salt, solvation sequence, thermoresponsive polymer

## Abstract

Understanding the salt effects on solvation behaviors of thermoresponsive polymers is crucial for designing and optimizing responsive systems suitable for diverse environments. In this work, the effect of potassium salts (CH_3_COOK, KCl, KBr, KI, and KNO_3_) on solvation dynamics of poly(4‐(*N*‐(3'‐methacrylamidopropyl)‐*N,N*‐dimethylammonio) butane‐1‐sulfonate) (PSBP), poly(*N*‐isopropylmethacrylamide) (PNIPMAM), and PSBP‐*b*‐PNIPMAM films is investigated under saturated water and mixed water/methanol vapor via advanced in situ neutron/optical characterization techniques. These findings reveal that potassium salts enhance the films' hygroscopicity or methanol‐induced swellability. Interestingly, the anions effects do not mirror the empirical Hofmeister series, which describes the salting‐in effects for such polymers in dilute aqueous solution, particularly evident in PSBP films with an approximately inverted order. PNIPMAM and PSBP‐*b*‐PNIPMAM exhibit pronounced deviations from such an inverted correlation and vary somewhat for water‐rich and methanol‐rich atmospheres. Molecular dynamics (MD) simulations suggest that the observed orders of solvation result from the accessibility of the hydrated solvation shells close to the PSBP‐*b*‐PNIPMAM chains.

## Graphical abstract

The effect of potassium salts (CH_3_COOK, KCl, KBr, KI, and KNO_3_) on solvation dynamics is investigated for poly(4‐(*N*‐(3‐methacrylamidopropyl)‐*N,N*‐dimethylammonio) butane‐1‐sulfonate) (PSBP), poly(*N*‐isopropylmethacrylamide) (PNIPMAM), and PSBP‐*b*‐PNIPMAM films under saturated water and mixed water/methanol vapor via advanced in situ neutron/optical characterization techniques. The anions effects do not mirror the empirical Hofmeister series.

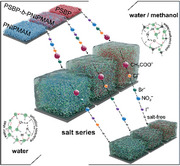

## 1 Introduction

Typically, the presence of salts affects the physicochemical properties of solutions as well as of solid systems in an ion‐specific way.^[^ ^1^ ^]^ In many cases, the anions exhibit a more pronounced effect than cations, following an empirical trend that is commonly known as the Hofmeister series, which was established according to their ability to promote the salting‐in or salting‐out of proteins in dilute aqueous solution^[^ ^1^, ^2^ ^]^:

(1) $$\begin{array}{ccc} \; & & C{O_{3}}^{2-}>S{O_{4}}^{2-}>\mathrm{OP}O_{3}H^{2-}>F^{-}\\ & & >CH_{3}\mathrm{CO}O^{-}>Cl^{-}>Br^{-}\approx N{O_{3}}^{-}\\ & & >I^{-}>\mathrm{Cl}{O_{4}}^{-}>\mathrm{SC}N^{-} \end{array}$$

While the explanation of the Hofmeister series is still controversial, the understanding of the (empirically found, purely descriptive) Hofmeister series has made important progress in the past two decades. Nevertheless, the understanding is far from complete, and a number of so far deviations from Hofmeister‐series‐based predictions are known that still await satisfactory explanations. The anions to the left side of Cl‾ are often referred to as kosmotropes (due to their presumed ability to stabilize the hydrogen bonding network between the water molecules), while the anions standing right of Cl‾ are referred to as chaotropes (due to their presumed ability to disrupt this network). The addition of kosmotropes reduces the hydration of proteins and most water‐soluble macromolecules and induces a salting‐out effect. Conversely, the presence of chaotropes enhances the hydration of proteins and most macromolecules, provoking a salting‐in effect. For the special case of zwitterionic poly(sulfobetaine)s that are thermoresponsive by showing upper critical solution temperature (UCST) behavior in water, their phase transition temperatures are increasingly reduced when adding salts with anions of increasingly chaotropic character.^[^ ^3^ ^]^

Whereas thermoresponsive polymers exhibiting a UCST transition seem to be rare in water,^[^ ^4^ ^]^ thermoresponsive behavior exhibiting a lower critical solution temperature (LCST) transition is widespread among non‐ionic water‐soluble polymers.^[^ ^5^ ^]^ Over the past decades, many studies investigated the specific ion effect for thermoresponsive polymers on the LCST behavior in aqueous solution.^[^ ^6^ ^]^ Among the numerous polymers investigated, poly(*N*‐isopropylacrylamide) (PNIPAM) is probably the best‐studied one.^[^ ^1^, ^6^, ^7^ ^]^ For example, Lopéz‐Léon et al.^[^ ^7d^ ^]^ reported that the LCST and particle size of PNIPAM nano‐gels are highly correlated with the Hofmeister series. Qian and coworkers ^[^ ^7c^ ^]^ showed a direct interaction between the cations and PNIPAM by molecular dynamics (MD) simulations and demonstrated that the anions shift the LCST down following the Hofmeister series from kosmotropes to chaotropes. Bergbreiter and co‐workers^[^ ^7a^ ^]^ further revealed that the effect of the anion on the LCST of PNIPAM can be ascribed to three types of interactions of anions with the polymer and its hydrating water molecules, including i) the hydrophobic hydration of the molecule associated with surface tension, ii) direct binding of the anion to the amide group, and iii) hydrogen bonding of the amide and its destabilization through polarization by the anion. More recently, due to the similarity of their hydrophilic and hydrophobic groups, poly(*N*‐isopropylmethacrylamide) (PNIPMAM) has been explored as an alternative to PNIPAM.^[^ ^8^ ^]^

Other than by adding salts, the thermoresponsiveness of polymers in water can also be modulated by adding organic co‐solvents. A particularity arises for the LCST behavior of PNIPAM as well as of PNIPMAM, which exhibits co‐nonsolvency for a number of polar solvents, including methanol as the most studied example,^[^ ^9^ ^]^ but also ethanol,^[^ ^10^ ^]^ acetone,^[^ ^11^ ^]^ dimethyl sulfoxide (DMSO),^[^ ^11^ ^]^ or dimethylformamide (DMF).^[^ ^12^ ^]^ Although all of those are good solvents for both polymers, their addition to water decreases the polymer solubility.^[^ ^10^, ^13^ ^]^ The reasons for co‐nonsolvency behavior are controversially discussed and seem complex, particularly as the term comprises several thermodynamic scenarios.^[^ ^13^ ^]^ In the simplest one, the transition in the ternary phase diagram between solubility in water via an insoluble region to solubility in the co‐solvent is due to an LCST transition that shows a minimum at intermediate contents of the co‐solvent in the mixture. In the second scenario, the sequence solubility – insolubility – solubility is characterized by the occurrence and steep drop of an LCST‐transition with increasing co‐solvent content resulting eventually in a region of insolubility. This is followed by the occurrence of a UCST transition that steeply falls with further increase of the co‐solvent content, thus re‐establishing the polymer's solubility.^[^ ^14^ ^]^ While the latter scenario seems to be the more widespread case, co‐nonsolvency of PNIPAM and PNIPMAM in water‐methanol mixtures follows the former scenario. A third thermodynamic scenario,^[^ ^4^ ^]^ namely co‐nonsolvency in combination with UCST‐behavior in both the water‐rich and the co‐solvent‐rich regimes, where the UCST becomes exceedingly high at intermediate co‐solvent contents, but is much lower or even absent in pure water and in the pure co‐solvent, has been observed only in exceptional cases up to now.^[^ ^15^ ^]^

Notwithstanding that the reasons for co‐nonsolvency are still under debate, a frequently proposed explanation evokes that the added co‐solvent disrupts (or enhances) the water structure. Accordingly, the addition of salts (be they kosmotropes or chaotropes) can be expected to modify the co‐nonsolvency (or co‐solvency) behavior of PNIPAM and PNIPMAM markedly.^[^ ^13^, ^16^ ^]^ Within this line of reasoning, Elaissari and coworkers,^[^ ^17^ ^]^ reported the specific effects of different anions on the cononsolvency behavior of PNIPAM microgels in water/ethanol mixtures. The addition of salts induced the particles to collapse, and promoted the system partial destabilization when the *V_ethanol_* (ethanol volume ratio) was <20%, while the system was driven to complete destabilization as *V_ethano_*_l_ >20%.

To the best of our knowledge, salt effects on co‐nonsolvency behavior of UCST‐type thermoresponsive polymers or on diblock copolymers (DBCs) that combine two thermoresponsive blocks with LCST‐ and UCST‐type behavior, have not been investigated yet, especially not in thin film geometry for polymer‐rich systems in a vapor environment. Addressing such a scenario, we select PNIPMAM as LCST‐type polymer, and poly(4‐(*N*‐(3‐methacrylamidopropyl)‐*N,N*‐dimethylammonio) butane‐1‐sulfonate) (PSBP) as UCST‐type polymer for our investigations, as the latter bears the poly(methacrylamide) backbone and hydrophilic amide groups in common with PNIPMAM. Also, salt effects on critical solution temperatures (CSTs) of PSBP and PSBP‐*b*‐PNIPMAM in dilute solutions have been investigated. For the homopolymer PSBP, various salts were identified to induce significant salting‐in effects on the UCST‐type cloud points in 5% wt.% aqueous solutions. The effectiveness of these salts follows the order (NH_4_)_2_SO_4_ ≈ Na_2_SO_4_ < NaCl < NaBr, aligning with the Hofmeister series.^[^ ^18^ ^]^ Notably, the influence of NaBr on both UCST and LCST transitions of PSBP_50_‐*b*‐PNIPMAM_155_ (which has different block lengths compared to the current study) was examined in a dilute solution at a concentration of 50 g L^−1^ polymer. This analysis revealed a substantial salting‐in effect on the PSBP block, with the clearing point decreasing by 50 °C upon heating. Conversely, no significant salt effect was observed on the cloud point of the PNIPMAM block at concentrations below 25 mm.^[^ ^15b^ ^]^ In addition, the co‐nonsolvency behavior of structurally closely related DBCs has already been addressed.^[^ ^15^, ^19^ ^]^

**Figure 1:**
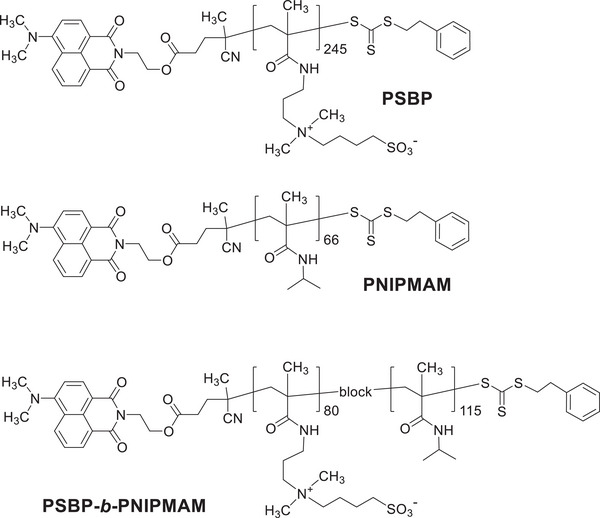
Chemical structure of the studied polymers PSBP, PNIPMAM, and PSBP‐*b*‐PNIPMAM. The chromophore‐labeled end groups are incorporated to support the molecular analysis and to determine the number of average molar masses *M_n_*, and the respective degrees of polymerization *DP_n_*.

Hence, in the present work, PSBP, PNIPMAM, and PSBP‐*b*‐PNIPMAM (**Figure** **1**) are chosen as representatives of UCST, LCST, and UCST‐*b*‐LCST type thermoresponsive polymers to examine the salt effect on the solvation behavior of thin films in pure water vapor and in mixed water/methanol vapor. We focus on the potassium salts CH_3_COOK, KCl, KBr, KI, and KNO_3_, whose anions cover a large part of the Hofmeister series from kosmotropic to chaotropic behavior. We use in situ spectral reflectance (SR), time‐of‐flight neutron reflectometry (ToF‐NR), and Fourier‐transform infrared (FT‐IR) spectroscopy to characterize the time‐resolved film thickness changes, the solvent contents, and the local polymer‐solvent interactions, respectively. To determine in which period most of the solvation events occur that respond to the water vapor, we use the 2D FT‐IR (2D FT‐IR) correlation analysis to study the first 30 FT‐IR spectra. Furthermore, we use MD simulations to discuss the accessibility of hydrated solvation shells in the case of the different salts.

## 2 Results and Discussion

The results include two sections. In the first section, the salt effects (CH_3_COOK, KCl, KBr, KI, and KNO_3_) on the film thickness (PSBP, PNIPMAM, and PSBP‐*b*‐PNIPMAM) are investigated by in situ SR measurements, responding to a stepwise exchanged vapor atmosphere starting with 100 vol% H_2_O vapor and ending with 100 vol% CH_3_OH vapor. In each step, 10 vol% of CH_3_OH vapor replaces successively H_2_O vapor as illustrated in Figure S1a (Supporting Information). In the second section, the salt effects on initial swelling in pure water vapor and subsequent rapid film contraction in a mixed water/methanol vapor are investigated further with in situ SR, ToF‐NR, and FT‐IR measurements. The applied vapor switching protocol is illustrated in Figure S1b (Supporting Information). To echo the study of swelling in pure methanol vapor in the first section, a further in situ SR measurement is performed following the vapor protocol in Figure S1c (Supporting Information). Besides, 2D FT‐IR is used to determine the perturbation sequence of hydration events responding to the pure water vapor, and MD simulations are used to observe the salt effect on accessibility of solvation shells and the conformation transition of PSBP‐*b*‐PNIPMAM chains.

### 2.1 Thin Film Response to a Stepwise Exchange of the Vapor Atmosphere

**Figure 2:**
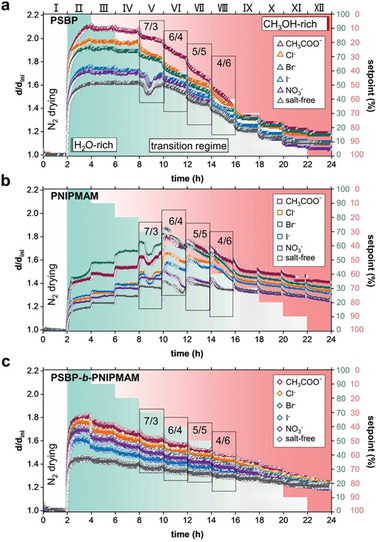
Evolution of the swelling ratio (*d/d_ini_*, i.e., the ratio of thickness at time *t*, *d*, to the initial thickness, *d_ini_*) of thin polymer films following a stepwise exchange of the surrounding vapor atmosphere obtained from SR measurements as a function of time for a) PSBP, b) PNIPMAM, and c) PSBP‐*b*‐PNIPMAM. In each step, 10 vol% of the original H_2_O vapor is replaced by CH_3_OH vapor, corresponding to the green and red setpoint on the right y‐axis, respectively. After the N_2_ drying (stage I), the H_2_O‐rich regime (stages II–IV) transforms into a CH_3_OH‐rich regime (stages IX–XII) through the transition regime (stages V–VIII, and indicated by black wireframes). The different colors of the symbols indicate the individual salts loaded into the films.

**Figure** **2** displays the swelling ratio as a function of time following a stepwise exchanged vapor protocol (Figure S1a, Supporting Information). Based on the relative mixing ratio, the process can be divided into three regimes (H_2_O‐rich, transition, and CH_3_OH‐rich). As shown in Figure 2, the overall trends of the swelling/deswelling behavior of the PSBP, PNIPMAM, and PSBP‐*b*‐PNIPMAM films basically depend on the vapor composition. The added salts amplify the swelling/deswelling behavior, but do not alter the overall trend. If the swelling ratio increases with added methanol, we classify the phenomena as a positive response and refer to it as a positive cosolvent effect (Figure 2b). In the opposite case (Figure 2a,c), we classify it as a negative response and refer to it as a negative cosolvent effect. Since the relative swelling/deswelling degree of each type of salt‐loaded sample depends on the type of anions, especially during the H_2_O‐rich and middle transition regimes, we compare the anion specificity of the effects with their position in the Hofmeister series, which is known to govern the solution behavior of such polymers. Furthermore, the phenomenon that the loaded salts enhance the swelling ratio is attributed to a salt‐induced solvation effect; otherwise, to a salt‐induced desolvation effect. Accordingly, the observed behavior is considered as an interplay between the cosolvent and salt effects. The interplay of the two effects within the three different regimes on the PSBP, PNIPMAM, and PSBP‐*b*‐PNIPMAM films is discussed below.

In the H_2_O‐rich regime (stages II–IV, where *V_methanol_* <30%), the swelling ratio remains almost constant for PSBP, while it increases for PNIPMAM and decreases for PSBP‐*b*‐PNIPMAM, respectively. This behavior indicates that at low *V_methanol_*, the added methanol influences the PSBP film only weakly, while it presents a positive cosolvent effect for PNIPMAM and a negative cosolvent effect for PSBP‐*b*‐PNIPMAM. Thus, in the H_2_O‐rich regime, the salt effect mainly dominates the swelling behaviors for the PSBP film, and a positive cosolvent effect results in enhanced swelling for the PNIPMAM film, while a negative cosolvent effect results in the collapse of the DBC film. A balance between the salt and cosolvent effects results in an equilibrated thickness for each stage.

The extent of the salt‐mediated equilibrated thickness values follows the order CH_3_COO‾ > Cl‾ > Br‾ > I‾ > NO_3_‾ in the case of PSBP, which approximately inverts the order of the Hofmeister series that governs salting‐in efficiency of these anions for PSBP in aqueous solution.^[^ ^18^, ^20^ ^]^ The corresponding orders of anions effects are Br‾ > CH_3_COO‾ > Cl‾ > I‾ > NO_3_‾ for PNIPMAM and CH_3_COO‾ > Cl‾ > Br‾ > NO_3_‾ > I‾ for the DBC. Hence, they also seem to follow, to a certain extent, an inverted Hofmeister correlation, but with polymer‐specific variations. Note, that the salt‐loaded films reach a higher thickness than corresponding salt‐free films, illustrating a salting‐in effect for each type of polymer, promoting water swelling, as addressed in previous studies.^[^ ^21^ ^]^ Besides, for the films loaded with the same anion or without salt, the equilibrated thicknesses at a specific mixing ratio (i.e., as a function of *V_methanol_*) are higher for PSBP‐*b*‐PNIPMAM than for PNIPMAM but lower than for PSBP. This finding indicates a stronger water absorption by PSBP films than by PNPMAM films of similar thickness.

During the transition regime (stages V–VIII, where 30% < *V_methanol_* < 70%), the swelling ratios undergo discrepant transitions for the PSBP and PNIPMAM films. Once *V_methanol_* exceeds 30%, all PSBP samples contract dramatically (however, at different rates). This observation implies that a negative cosolvent effect emerges and overrides the salt effect since no thickness plateau is reached responding to a stepwise exchange in 2 h. Nevertheless, the decreased swelling ratios still obey the salt series that are found in the H_2_O‐rich regime.

Differently, when *V_methanol_* exceeds 40%, all PNIPMAM films undergo a continuous contraction with a short inevitable re‐swelling, which starts when transitioning between adjacent stages (i.e., *V_methanol_* instantaneously increases) and is unobtrusive in the PSBP and DBC films. Interestingly, once *V_methanol_* reaches 40% (VI), the Br‾‐loaded PNIPMAM film collapses rapidly after the reswelling, causing a pass down of swelling ratio over the CH_3_COO‾‐loaded film, whereas the Cl‾‐loaded film reswells more, causing a surpass of swelling ratio over the I‾‐loaded film. As a result, the order of the anion‐mediated effect in the transition regime is changed for PNIPMAM films from Br‾ > CH_3_COO‾ > Cl‾ > I‾ > NO_3_‾ to CH_3_COO‾ > Br‾ > Cl‾ > I‾ > NO_3_‾. Therefore, we conclude that the positive cosolvent effect is transformed into the negative cosolvent effect as *V_methanol_* exceeds 40%, and afterward, the negative cosolvent effect overrides the salt effect since no thickness plateau is reached responding to a stepwise exchange in 2 h.

Even so, the transient positive cosolvent effect caused by a sudden *V_methanol_* increase is not shieldable during the transition regime (stages V‐VIII). Notably, part of the PSBP (I‾‐, NO_3_‾‐loaded and salt‐free) and PNIPMAM (Br‾‐, CH_3_COO‾‐, Cl‾‐ and I‾‐loaded) curves suffer from a downward inverted peak in stage V, but basically return to the degree of swelling before. In contrast, the DBC films collapse stepwise and synchronously at a similar rate as produced in stages III and IV, and continue to follow the salt series of the H_2_O‐rich regime. Besides, concerning an approximate equilibrium of thickness at each stage, we conclude that although the negative cosolvent effect dominates the entire regime, a balance with the salt effect exists at each stage, which enables a near‐linear collapse with control of the thickness by the loaded specific anion species (Figure S2, Supporting Information).

During the CH_3_OH‐rich regime (stages IX–XII, where *V_methanol_* >70%), all PSBP films pursue the trend of shrinking thickness from the transition regime. This can even lead to partial overlap or crossover of the swelling ratios for different loaded salts. As a result, the order of the salt‐induced effects weakly changes to CH_3_COO‾ > Cl‾ ≈ Br‾ > I‾ ≈ NO_3_‾, i.e., the effects induced by Cl‾ and Br‾ become nearly the same. Differently, all PNIPMAM samples collapse nearly simultaneously by inheriting the trends (i.e., approximately constant differences among swelling ratios) seen in the stages before, and continue to follow the initial anion series. All DBC films continue the near‐linearly contraction, promoting swelling ratios to coincide at one point. Thus, for the DBC films, the changed salt series observed in the transition regime still remains, but the distinction is no longer significant in pure methanol vapor. Since all salt‐loaded samples present a stepwise equilibrated thickness, we conclude that at high *V_methanol_*, the negative cosolvent effect dominates the swelling behavior. Interestingly, the hitherto observed salt‐induced solvation effect of NO_3_‾ for PSBP and PNIPMAM is replaced in pure methanol vapor by a weak salt‐induced desolvation effect.

In a 2D geometry hydrogel where the polymer is the majority component, the solvent composition significantly influences polymer‐ion interactions, leading to solvation behavior that deviates from the traditional Hofmeister series observed in aqueous solutions. Particularly in zwitterionic PSBP films, non‐specific ion interactions with polymer ionic groups also alter the expected Hofmeister ordering, affecting solvent absorption, and polymer chain conformation changes.

### 2.2 Thin Film Hydration and Switching Kinetics

**Figure 3:**
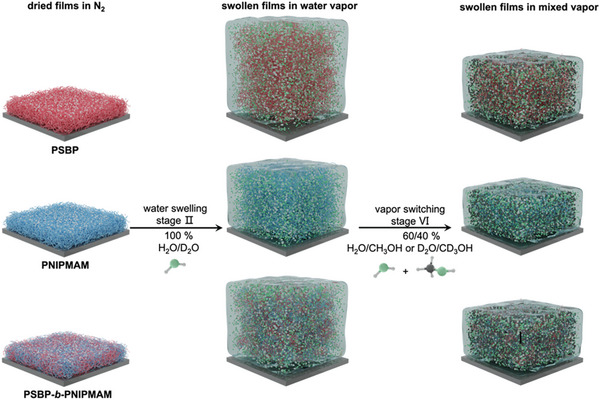
Experimental protocol applied for SR, ToF‐NR, and FT‐IR studies on PSBP, PNIPMAM, and PSBP‐*b*‐PNIPMAM thin films. Two kinetic processes are labeled: water swelling ((H_2_O or D_2_O = 100%, stage II) and vapor switching (H_2_O/CH_3_OH or D_2_O/CD_3_OH = 60/40%, stage VI).

For each polymer, the present study highlights a notable salt effect in stage II. Furthermore, a strong negative cosolvent effect in stage VI for PNIPMAM is observed, where the mixing ratio of H_2_O/CH_3_OH is 6/4. To further study the salt effect on the solvation behavior in H_2_O and in a subsequently mixed H_2_O/CH_3_OH vapor (i.e., upon the negative cosolvent effect), as well as the salt effect on the polymer‐solvent interactions, in situ SR, ToF‐NR, and FT‐IR measurements are conducted to characterize the time‐resolved film thickness changes, the solvent contents, and the local polymer‐solvent interactions. The experimental protocol is illustrated in **Figure** **3**.

#### 2.2.1 Kinetics of the Swelling Behavior

**Figure 4:**
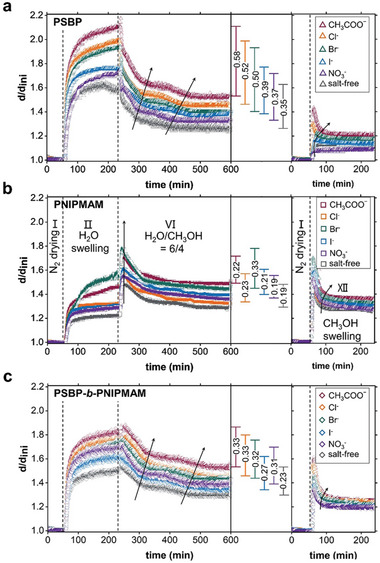
Evolution of the swelling ratio *d/d_ini_* obtained from SR measurements as a function of time for a) PSBP, b) PNIPMAM, and c) PSBP‐*b*‐PNIPMAM thin films with the stages: N_2_ drying (I), H_2_O swelling (II), vapor switching (H_2_O/CH_3_OH = 6/4, VI), and CH_3_OH swelling (XII). The largest absolute changes in swelling ratio at stage VI are shown with scale bars. The black arrows indicate the positions where the swelling ratios change.

All samples are investigated by in situ SR measurements upon H_2_O hydration and the subsequent H_2_O/CH_3_OH switching process. To echo the study in a CH_3_OH‐rich regime, further exploration of the salt effect in pure methanol vapor is performed following the vapor exchange protocol in Figure S1c (Supporting Information). In agreement with the findings at stage II (see Figure 2), once exposed to an H_2_O vapor, all samples swell strongly to a defined threshold (**Figure** **4**), depending on the loaded salt, which differs in the anion species. Furthermore, the swelling ratios of each type of polymer thin film obey the salt series introduced in the H_2_O‐rich regime as discussed above. Again, the loaded salts provoke a salt‐induced solvation effect, enhancing the water absorption.

In mixed H_2_O/CH_3_OH vapor (stage VI), all samples contract but undergo several distinguishable multistep transitions depending on the polymer type. As illustrated in Figure 4a, all PSBP films undergo a spontaneous collapse within the first 5 min, followed by a nearly linear contraction period (50–70 min). Afterward, they contract slowly to a final equilibrated state. Compared to the stepwise collapses at stages V and VI in Figure 2a, the CH_3_COO‾, Cl‾, and Br‾‐loaded PSBP films tend to reproduce the two‐step variations to a larger extent in a shorter time. Importantly, except for the changes in swelling ratio, both the duration of the middle near‐linear or subsequent slow contraction, as marked by the black arrows, also obey the original order that resembles an inverted Hofmeister series. Conversely, all PNIPMAM films reswell strongly in the first 20 min of stage VI due to the positive cosolvent effect, but then contract slowly due to the negative cosolvent effect until a plateau is reached, as shown in Figure 4b. Thus, a positive cosolvent effect coexists with a negative cosolvent effect. Moreover, the order of the anion effects encountered in pure H_2_O vapor is no longer followed (i.e., Cl^−^ loses its priority position over I^−^ and NO_3_^−^ in mixed H_2_O/CH_3_OH vapor).

For all DBC films in Figure 4c, not only a weak reswelling is seen during the first 7 min of stage VI, but also the two‐step collapse is found (weaker than PSBP and stronger than PNIPMAM). Thus, the behavior of the DBC films upon vapor switching shows superposed contributions of both the PSBP and PNIPMAM blocks. Interestingly, in this switching experiment, the anion effects follow the order CH_3_COO‾ > Cl‾ > Br‾ > NO_3_‾ > I‾, i.e., it mirrors the Hofmeister series. Again, compared to the salt‐free samples, the higher swelling ratio for salt‐loaded samples illustrates a significant salt‐induced solvation effect even in a mixed H_2_O/CH_3_OH atmosphere.

In pure CH_3_OH vapor (stage XII), although all films are slightly swollen, the salt effects are very small (the swelling ratios do not differ more than 0.17 for PSBP, 0.14 for PNIPMAM, and 0.10 for PSBP‐*b*‐PNIPMAM). Unlike the swelling behavior in pure H_2_O vapor, all films swell instantaneously in CH_3_OH vapor to a high ratio and then contract rapidly to their equilibrium thickness. This finding implies that the films experience a process of instantaneous absorption and subsequently, rapid release of methanol molecules, which might be the reason for the positive cosolvent effect. Obviously, the relaxation time and thickness changes do not depend only on the anion species, but also on the polymer type (with PNIPMAM > DBC > PSBP). Despite the difficulty in distinguishing the anion‐specific effects due to partial overlapping or interspersing, the salt series in pure CH_3_OH vapor are determined as: CH_3_COO‾ > Cl‾ > Br‾ > I‾ > NO_3_‾ for PSBP, CH_3_COO‾ > Br‾ > Cl‾ > I‾ > NO_3_‾ for PNIPMAM and CH_3_COO‾ > Cl‾ > Br‾ ≈ I‾ > NO_3_‾ for the DBC. Again, a weak salt‐induced desolvation effect of NO_3_‾ is observed in pure CH_3_OH vapor for NO_3_‾‐loaded PSBP and DBC films.

#### 2.2.2 Kinetics of Solvent Absorption

**Figure 5:**
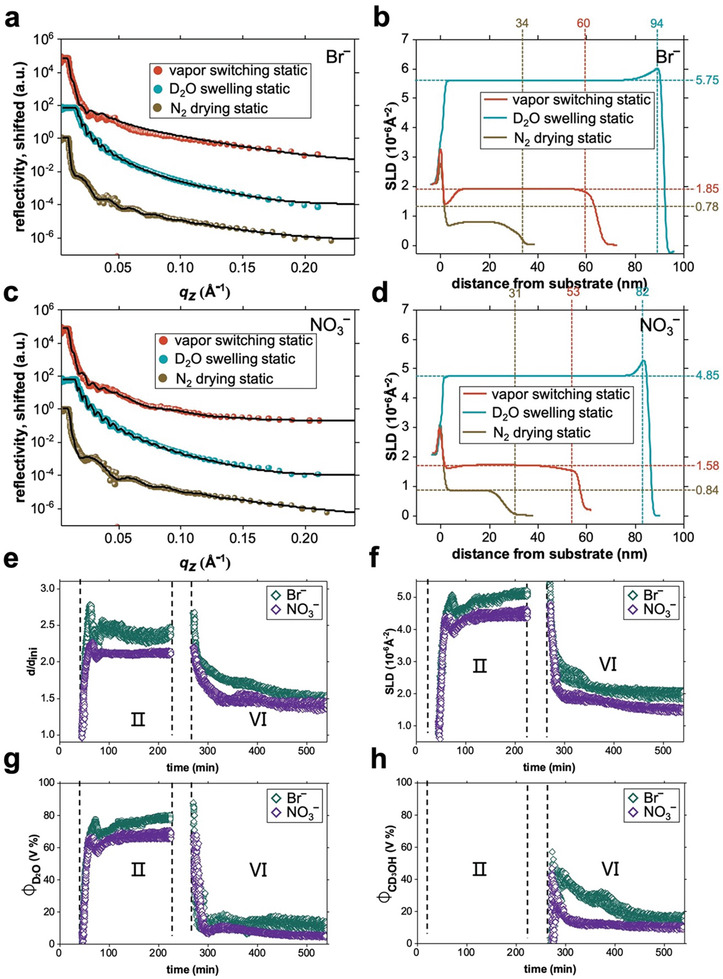
ToF‐NR data of Br‾‐ and NO_3_‾‐ loaded PSBP‐*b*‐PNIPMAM thin films: a–d) static data and e–h) kinetic data. a,c) StaticToF‐NR data (symbols) and b,d) determined SLD profiles. The static NR data at the N_2_ drying state (I), D_2_O swelling state (II), and vapor switching state (VI) are colored in brown, dark teal, and red, respectively. The best fits obtained via a three‐layer model are presented as black solid lines in a,c). The thickness and SLD values are listed on the corresponding axis in (b,d). Evolution of the e) swelling ratio *d/d_ini_*, f) obtained bulk SLD, g) calculated D_2_O and f) CD_3_OH contents as a function of time at two stages: D_2_O swelling (II) and vapor switching (D_2_O/CD_3_OH = 6/4, VI), colored in green and purple for Br‾‐ and NO_3_‾‐loaded films respectively.

With static and kinetic ToF‐NR measurements we examine the solvent absorption, as presented in **Figure** **5**. Due to the limited availability of neutron beamtime, we focus on the Br‾ and NO_3_‾‐loaded DBC films. Moreover, D_2_O and CD_3_OH are selected to enhance the scattering length density (SLD) contrast for the neutron scattering experiment. In contrast, the film thicknesses are chosen such that they are appropriate for ToF‐NR (34 nm for Br‾ and 31 nm for NO_3_‾‐loaded). The static NR curves with the best fits, as well as the obtained SLD profiles, are shown in Figure 5a–d. A three‐layer model, consisting of a substrate‐polymer interface, a bulk polymer layer, and a polymer‐air interface, is used to fit the ToF‐NR data. Table S1 (Supporting Information) presents the calculated theoretical SLD values of all involved materials. The method to calculate the SLD of the polymers is described in the “ToF‐NR” section in the Supporting Information.

Comparing the NR curves between N_2_ drying static and D_2_O swelling static (Figure 5a,c), the Kiessig fringe spacing decreases, and the critical edge shifts toward higher *q_z_* values, illustrating the increase in film thickness and SLD values (i.e., the incorporated D_2_O content), as seen in the corresponding SLD profiles (Figure 5b,d). In contrast, the corresponding SLD profiles of vapor‐switching static curves demonstrate a decrease in both thickness and SLD values (Figure 5b,d). Following Equations S4–S7 (Supporting Information), the absorbed solvent content (D_2_O and CD_3_OH) is calculated and given in Table S2 (Supporting Information). In agreement with the SR results, the Br‾‐loaded DBC film not only realizes a higher swelling ratio and a larger D_2_O absorption in both scenarios compared to the NO_3_‾‐loaded DBC film, but also has a larger CD_3_OH content in stage VI.

Figure 5e–h presents the findings from the kinetic ToF‐NR data analysis of both salt‐loaded DBC samples. As expected, swelling ratio, SLD values, and solvent contents (D_2_O and CD_3_OH) are higher for the Br‾‐loaded DBC film than for the NO_3_‾‐loaded one. Analogous to the SR results, the effect of the presence of Br‾ on the solvation behavior of the DBC film is stronger than NO_3_‾, matching the observed salt series as discussed before. Upon D_2_O hydration (stage II), a synchronous fluctuation that appears in the swelling ratio, SLD profiles, and D_2_O content is ascribed to the rearrangement of polymer chains induced by the rapid water absorption.^[^ ^15^, ^19^, ^21^ ^]^

Attractively, upon vapor switching (stage VI), the two DBC films exhibit an asynchronous variation in the CD_3_OH content, while a synchronous variation in the D_2_O content is found (Figure 5c,d). Once the mixed D_2_O/CD_3_OH vapor replaces the D_2_O vapor, a fast release of D_2_O molecules accompanied by rapid absorption of CD_3_OH molecules is observed in 10–15 min. In the following 70 min before reaching the final equilibrium, the CD_3_OH content of the Br‾‐loaded film decreases more slowly than for the NO_3_‾‐loaded film, while both their D_2_O contents increase slightly. This behavior explains why the Br‾‐loaded film presents a higher swelling ratio than the NO_3_‾‐loaded one at the first near‐linear contraction in Figures 4c and 5e. Considering the large difference between both CD_3_OH contents and the small difference between both D_2_O contents, we infer that the presence of the anions mainly affects the methanol absorption, resulting in different film thicknesses during this stage of the kinetic process.

#### 2.2.3 Salt‐Induced Interactions Between Polymer and Solvent

**Figure 6:**
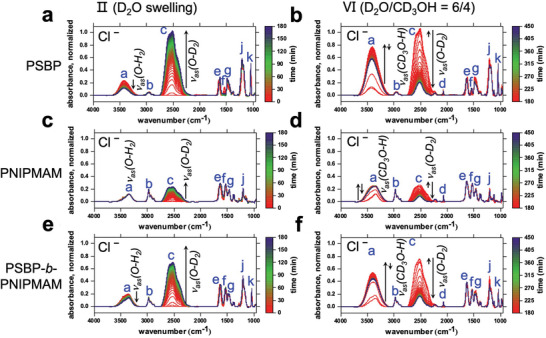
FT‐IR spectra of Cl‾‐loaded thin films of a,b) PSBP, c,d) PNIPMAM, and e,f) PSBP‐*b*‐PNIPMAM, upon a,c,e) D_2_O swelling (stage II), and b,d,f) subsequent vapor switching (D_2_O/CD_3_OH = 6/4, stage VI). The characteristic bands listed in Table S3 (Supporting Information) are indicated by blue lowercase letters. The black arrows highlight the growth or decay of characteristic signals.

FT‐IR spectroscopy is performed to track the formation or reduction of solvation shells on a molecular level. Again, the deuterated solvents D_2_O and CD_3_OH are selected to avoid the overlap of specific IR bands. The prepared films are thicker than in the previous experiments, with thicknesses of ≈4–6 µm, to ensure sufficient intensity of the IR signals. The FT‐IR spectra are exemplified in **Figure** **6** for the Cl‾‐loaded films, to explain the observed changes. For clarity, Figures S4—S9 (Supporting Information) present the resulting full, enlarged FT‐IR spectra of all PSBP, PNIPMAM, and DBC samples. Besides, all FT‐IR bands assigned to polymers are listed in Table S3 (Supporting Information).

Upon D_2_O hydration (Figure 6a,c,e), the height and area of the hydroxyl band of D_2_O (*ν_as_(O‐D_2_)* ≈ 2540 cm^−1^) increase while they decrease for the one of H_2_O (*ν_as_(O‐H_2_)* ≈ 3400 cm^−1^). This observation indicates that the absorbing D_2_O molecules replace the residual H_2_O being already present in the films. Analogously, upon vapor switching, the reversed changes of both hydroxyl bands illustrate the concomitant D_2_O release and CD_3_OH absorption. The emergence of a peak d (*v_as_(C‐D_3_OH)*, specifically attributed to the CD_3_OH) corroborates the absorption of CD_3_OH. As seen in the enlarged spectra of Cl‾‐loaded PSBP (marked by black arrows in Figure S7c, Supporting Information), the amide I band shifts toward lower wavenumbers as the carbonyl groups act as hydrogen‐bond acceptors, while the amide II band disappears accompanied by an increase of the amide II׳ band due to an H/D exchange (N‐H to N‐D) upon D_2_O hydration. The introduction of CD_3_OH prompts the reverse change, as shown in Figure S7d (Supporting Information).

**Figure 7:**
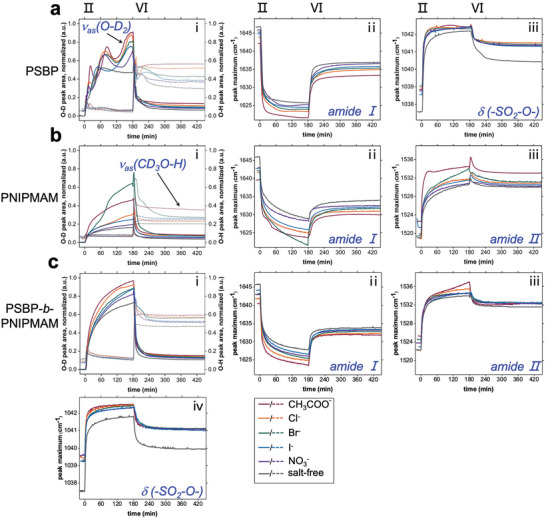
Temporal evolution of normalized peak areas of O‐D and O‐H stretching vibrations, and peak positions of the amide I band, amide II band, and the symmetric S‐O stretching vibration for a) PSBP, b) PNIPMAM, and c) PSBP‐*b*‐PNIPMAM thin films. The peak areas of O‐H stretching vibrations in Figure “i” are plotted as dotted lines corresponding to the right y‐axis, to distinguish them from the peak areas of the O‐D stretching vibrations that are plotted as continuous lines corresponding to the left y‐axis.

Differently, the amide II band shifts toward higher wavenumbers for Cl‾‐loaded films of PNIPMAM (Figure S8c, Supporting Information) and then shifts reversely (Figure S8d, Supporting Information), as the amide protons act as hydrogen‐bond donors. An increase followed by an equivalent decrease in the amide II׳ signal (Figure S8d, Supporting Information) indicates an H/D exchange provoked by the absorbed CD_3_OH molecules, fading out over time. As marked by the black arrows in Figure S9c,d (Supporting Information), the variations in the amide II and II׳ bands positions and heights of the DBC films result from superposed contributions of the two blocks. The findings described above also apply to other salt‐loaded samples for each type of polymer. Besides, the FT‐IR peaks resulting from CH_3_COO‾ (*v* ≈ 1590–1550 cm^−1^) and NO_3_‾ (*v* ≈ 1410–1370 cm^−1^) are marked in the static FT‐IR spectra (Figure S10, Supporting Information). Although the absorbed D_2_O and CD_3_OH contents can be partly distinguished via their hydroxyl bands in the static FT‐IR spectra, it remains challenging to determine a trend for the salt series. The observations from the kinetic FT‐IR study are plotted in **Figure** **7**, including the temporal evolution of the relative D_2_O and CD_3_OH contents (Figure 7a‐i,b‐i,c‐i) as well as the peak shifts of characteristic signals (Figure 7a‐ii,iii,b‐ii,iii,c‐ii,iii,iv).

Upon D_2_O hydration, the trend of the salt effects on the absorbed D_2_O amount varies somewhat between the different polymer films, in agreement with the observations by the SR measurements (see Figure 2). While the order of the effects may be formally described by an inverted Hofmeister series for the DBC (CH_3_COO‾ > Cl‾ > Br‾ > NO_3_‾ > I‾) and ‐at least approximately‐ also for PSBP (CH_3_COO‾ > Cl‾ > Br‾ > I‾ > NO_3_‾), the deviations from such an analogy are rather pronounced for PNIPMAM (Br‾ > CH_3_COO‾ > Cl‾ > I‾ > NO_3_‾). The three consecutive steps of D_2_O absorption in the PSBP films (Figure 7a‐i) are attributed to the strong water absorption ability of the polyzwitterion, which might cause a mismatch between the water absorption rates and polymer chain conformation changes. Upon vapor switching, the salt effects are easily determined from the equilibrated CD_3_OH contents (stage VI in Figure 7a,d,c‐i). The determined salt series from the D_2_O uptake behavior remains unchanged in the CD_3_OH uptake in the presence of nearly equal amounts of D_2_O content, in agreement with findings from ToF‐NR. The short reswelling process during the first 10 min of stage XII is also clearly observed in the CD_3_OH content.

To reveal the salt effects on the local chemical surroundings, a Gauss fit is performed to FT‐IR data to detect the peak shifts of the characteristic signals (amide I band and anionic SO_3_^−^ group for PSBP, amide I and II bands for PNIPMAM and all three bands for DBC). For the amide bands, either the peak shifts upon D_2_O hydration or the subsequent backshifts upon vapor switching, follow the same trends identified before as a function of salt type. Interestingly, the shift of the amide II band of PNIPMAM films at stage VI (Figure 7b‐iii) features a small overshoot and is partially reversed to reach a similar position as upon D_2_O hydration. This observation coincides with the increase in height and area of the amide II׳ band in Figure S8 (Supporting Information), which is followed by an equivalent decrease subsequently.

As no further shift appears for the amide I band (Figure 7b‐ii), we infer that the reswelling process (positive cosolvent effect) in mixed D_2_O/CD_3_OH vapor is due to an H/D exchange at the amide group. Besides, for the Br‾‐loaded PNIPMAM film, an up/down change that makes the Br‾‐loaded curve crossing the CH_3_COO‾‐loaded curve is not observed in the amide II shift. This finding implies that the solvation shells at the amide I band mainly affect the PNIPMAM contraction. As for the peak of the anionic SO_3_^−^ group (Figure 7a‐iii,c‐iv) which is exclusive for the side chains of PSBP, the loaded salts push its initial position toward higher wavenumbers as a general effect, while reducing the peak shift compared to the salt‐free control. However, no specific salt effect is observed. This finding suggests indiscriminate ionic interactions between SO_3_^−^ and loaded K^+^, which impairs the ability of the SO_3_^−^ group to act as a hydrogen‐bond acceptor. In all cases, a significant salt‐induced solvation effect is observed in the FT‐IR data of each type of polymer thin film, including solvent contents and peak shifts.

#### 2.2.4 Salt Effect on the Temporal Sequence of Solvation Events

**Figure 8:**
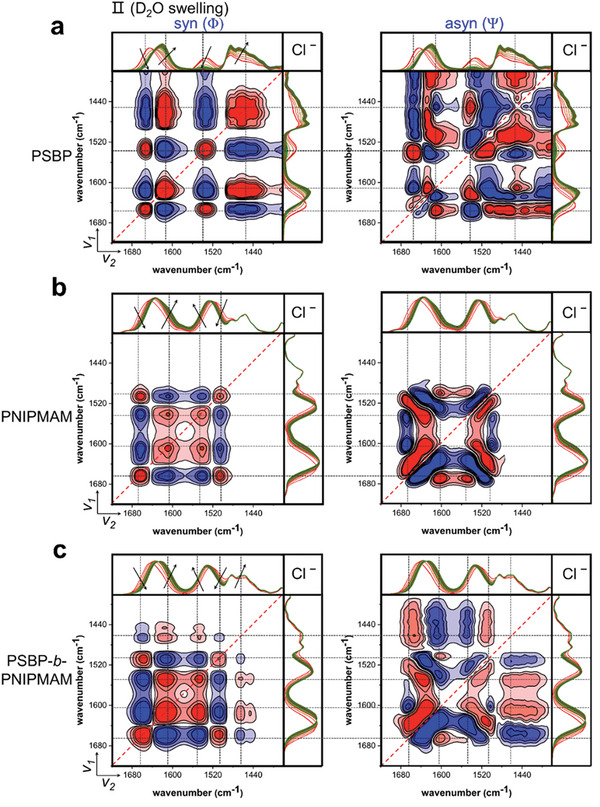
2D FT‐IR correlation contour maps of Cl‾‐loaded a) PSBP, b) PNIPMAM, and c) PSBP‐*b*‐PNIPMAM thin films upon D_2_O swelling process (stage II) in the region of 1720–1380 cm^−1^. Synchronous and asynchronous maps are marked as Φ and Ψ respectively, and two independent wavenumber axes are set as *v_1_* and *v_2_* (*v_1_* refers to the y‐axis and *v_2_* refers to the x‐axis). The black arrows highlight the fluctuation in spectral intensity. Positive correlation and negative correlation are colored in red and blue respectively.

**Figure** **8** presents the 2D FT‐IR correlation contour maps of the different Cl‾‐loaded polymer samples during the first 45 min upon D_2_O hydration, during which period most of the solvation events occur that respond to the D_2_O vapor, as marked by black arrows in the spectra on top of the synchronous maps. The observed decrease of the amide I signal is attributed to the breaking of intermolecular hydrogen bonds (C≐O···H─N), while the observed increase is attributed to the generation of amide I‐D_2_O hydrogen bonds (C≐O···D─O─D). Similarly, the increase in the amide II band in PNIPMAM and DBC films (Figure 8b,c) is attributed to the generation of amide II‐D_2_O hydrogen bonds (N─H···O─D_2_), while the decrease is attributed to the breaking of intermolecular hydrogen bonds (N─H···O≐C). In agreement with the findings from the FT‐IR spectra, the amide II fluctuation in PSBP (Figure 8a) is attributed to the generation of amide II‐D_2_O hydrogen bonds (N─H···O─D_2_) and a H/D exchange, while the latter one contributes to the increase in amide II׳ band (N─D···O─H/D). Note that the 2D plot only reveals the spectral fluctuation hidden by peak shift, but not the intensity change. Thus, the event that occurred at the amide II band in PSBP only represents the generation of N─H···O─D_2_ bonds, whereas the one that occurred at the amide II׳ band represents a H/D exchange. Corresponding to the described solvation events, strong autopeaks develop in the respective synchronous map diagonals.

As shown in the off‐diagonal region (*v_1_* > *v_2_*, Figure 8), the cross‐peaks are marked by gray dotted lines in both synchronous and asynchronous maps. According to Noda's rule^[^ ^22^ ^]^ and the analysis of Cl‾‐loaded samples described in the Supporting Information, the concluded sequence of solvation events is depicted as 1545 > 1620 > 1450 > 1655 cm^−1^ for Cl‾‐loaded PSBP film. It is depicted as 1605 > 1542 > 1665, 1500 cm^−1^ for Cl‾‐loaded PNIPMAM film, and depicted as 1605 > 1545 > 1667, 1505 > 1465 cm^−1^ for Cl‾‐loaded DBC film. For a comparative analysis, two types of contour maps of all salt‐loaded and salt‐free PSBP (Figure S11, Supporting Information), PNIPMAM (Figure S12, Supporting Information), and DBC (Figure S13, Supporting Information) samples are plotted in the Supporting Information. The salt effect on the temporal sequences of the assigned solvation events is summarized in **Table** **1** for all samples. As at stage VI, each sample undergoes multi‐responses to this vapor switching step as described by the normalized peak areas of the group‐specific IR‐bands (Figure 7a,b,c‐i), resulting in a disordered separation and distribution of cross‐peaks in the asynchronous maps. Therefore, 2D FT‐IR correlation analysis is not used to analyze the solvation sequence at stage VI.

**Table 1:** Temporal sequence of the solvation events responding to the exposure to D_2_O vapor (stage II).

Temporal sequence of solvation events in amide bands of PSBP, PNIMAM, PSBP‐b‐PNIPMAM						
PSBP	D_2_O swelling	Strong autopeaks				
		Amide I		Amide II	Amide II′	
		ν_as_(C = O⋅⋅⋅H‐N)	ν_as_(C = O⋅⋅⋅D‐O‐D)	δ (N‐H⋅⋅⋅O‐D_2_)	δ (N‐D⋅⋅⋅O‐H/D)	
		≈1655 cm^−1^	≈1620 cm^−1^	≈1545 cm^−1^	≈1450 cm^−1^	
	CH_3_COO‾	1560 >1620 > 1450 > 1545, 1655 CH_3_COO‾•D_2_O >ν_as_(C = O⋅⋅⋅D‐O‐D) > δ (N‐D⋅⋅⋅O‐H/D) > δ (N‐H⋅⋅⋅O‐D_2_), ν_as_(C = O⋅⋅⋅H‐N)				
	Cl‾	1545 > 1620 > 1450 > 1655 δ (N‐H⋅⋅⋅O‐D_2_) > ν_as_(C = O⋅⋅⋅D‐O‐D) > δ (N‐D⋅⋅⋅O‐H/D) > ν_as_(C = O⋅⋅⋅H‐N)				
	Br‾	1545 > 1620 > 1450 > 1655 δ (N‐H⋅⋅⋅O‐D_2_) > ν_as_(C = O⋅⋅⋅D‐O‐D) > δ (N‐D⋅⋅⋅O‐H/D) > ν_as_(C = O⋅⋅⋅H‐N)				
	I‾	1545 > 1620 > 1450 > 1655 δ (N‐H⋅⋅⋅O‐D_2_) > ν_as_(C = O⋅⋅⋅D‐O‐D) > δ (N‐D⋅⋅⋅O‐H/D) > ν_as_(C = O⋅⋅⋅H‐N)				
	NO_3_‾	1545, 1620 > 1450 > 1655 δ (N‐H⋅⋅⋅O‐D_2_), ν_as_(C = O⋅⋅⋅D‐O‐D) > δ (N‐D⋅⋅⋅O‐H/D) > ν_as_(C = O⋅⋅⋅H‐N)				
	Salt‐free	1545 > 1620 > 1450 > 1655 δ (N‐H⋅⋅⋅O‐D_2_) > ν_as_(C = O⋅⋅⋅D‐O‐D) > δ (N‐D⋅⋅⋅O‐H/D) > ν_as_(C = O⋅⋅⋅H‐N)				
PNIPMAM	D_2_O swelling	Strong autopeaks				
		Amide I		Amide II		Amide II′
		ν_as_(C = O⋅⋅⋅H‐N)	ν_as_(C = O⋅⋅⋅D‐O‐D)	δ (N‐H⋅⋅⋅O‐D_2_)	δ (N‐H⋅⋅⋅O = C)	δ (N‐H⋅⋅⋅O‐H/D)
		≈1665 cm^−1^	≈1605 cm^−1^	≈1542 cm^−1^	≈1500 cm^−1^	≈1460cm^−1^
	CH_3_COO‾	1460 > 1542 > 1605 > 1665, 1500 δ (N‐H⋅⋅⋅O‐H/D) > δ (N‐D⋅⋅⋅O‐D_2_) > ν_as_(C = O⋅⋅⋅D‐O‐D) > ν_as_(C = O⋅⋅⋅H‐N) > δ (N‐H⋅⋅⋅O = C)				
	Cl‾	1605 > 1542 > 1665, 1500 ν_as_(C = O⋅⋅⋅D‐O‐D) > δ (N‐H⋅⋅⋅O‐D_2_) > ν_as_(C = O⋅⋅⋅H‐N), δ (N‐H⋅⋅⋅O = C)				
	Br‾	1460 > 1542, 1605 > 1665 δ (N‐H⋅⋅⋅O‐H/D) > δ (N‐H⋅⋅⋅O‐D_2_), ν_as_(C = O⋅⋅⋅D‐O‐D) > ν_as_(C = O⋅⋅⋅H‐N)				
	I‾	1605 > 1542 > 1665, 1500 ν_as_(C = O⋅⋅⋅D‐O‐D) > δ (N‐H⋅⋅⋅O‐D_2_) > ν_as_(C = O⋅⋅⋅H‐N), δ (N‐H⋅⋅⋅O = C)				
	NO_3_‾	1605 > 1542 > 1665, 1500 ν_as_(C = O⋅⋅⋅D‐O‐D) > δ (N‐H⋅⋅⋅O‐D_2_) > ν_as_(C = O⋅⋅⋅H‐N), δ (N‐H⋅⋅⋅O = C)				
	Salt‐free	1460 > 1665, 1500 > 1605, 1542 δ (N‐H⋅⋅⋅O‐H/D) > ν_as_(C = O⋅⋅⋅H‐N), δ (N‐H⋅⋅⋅O = C) > ν_as_(C = O⋅⋅⋅D‐O‐D), δ (N‐H⋅⋅⋅O‐D_2_)				
PSBP‐b‐PNIPMAM	D_2_O swelling	Strong autopeaks				
		Amide I		Amide II		Amide II′
		ν_as_(C = O⋅⋅⋅H‐N)	ν_as_(C = O⋅⋅⋅D‐O‐D)	δ (N‐H⋅⋅⋅O‐D_2_)	δ (N‐H⋅⋅⋅O = C)	δ (N‐D⋅⋅⋅O‐H/D)
		≈1667 cm^−1^	≈1605 cm^−1^	≈1545 cm^−1^	≈1505 cm^−1^	≈1465cm^−1^
	CH_3_COO‾	1505 > 1605, 1667 > 1580, 1545,1465 δ (N‐H⋅⋅⋅O = C) > ν_as_(C = O⋅⋅⋅D‐O‐D), ν_as_(C = O⋅⋅⋅H‐N) > CH_3_COO‾•D_2_O, δ (N‐H⋅⋅⋅O‐D_2_), δ (N‐D⋅⋅⋅O‐H/D)				
	Cl‾	1605 > 1545 > 1667, 1505 > 1465 ν_as_(C = O⋅⋅⋅D‐O‐D) > δ (N‐H⋅⋅⋅O‐D_2_) > ν_as_(C = O⋅⋅⋅H‐N), δ (N‐H⋅⋅⋅O = C) > δ (N‐D⋅⋅⋅O‐H/D)				
	Br‾	1605 > 1545> 1667, 1505 > 1465 ν_as_(C = O⋅⋅⋅D‐O‐D) > δ (N‐H⋅⋅⋅O‐D_2_) > ν_as_(C = O⋅⋅⋅H‐N), δ (N‐H⋅⋅⋅O = C) > δ (N‐D⋅⋅⋅O‐H/D)				
	I‾	1605 > 1545 > 1667, 1505 > 1465 ν_as_(C = O⋅⋅⋅D‐O‐D) > δ (N‐H⋅⋅⋅O‐D_2_) > ν_as_(C = O⋅⋅⋅H‐N), δ (N‐H⋅⋅⋅O = C) > δ (N‐D⋅⋅⋅O‐H/D)				
	NO_3_‾	1605 > 1400,1545 > 1667, 1505 > 1465 ν_as_(C = O⋅⋅⋅D‐O‐D) > NO_3_‾•D_2_O, δ (N‐H⋅⋅⋅O‐D_2_) > ν_as_(C = O⋅⋅⋅H‐N), δ (N‐H⋅⋅⋅O = C) > δ (N‐D⋅⋅⋅O‐H/D)				
	Salt‐free	1605 > 1545 > 1667, 1505 > 1465 ν_as_(C = O⋅⋅⋅D‐O‐D) > δ (N‐H⋅⋅⋅O‐D_2_) > ν_as_(C = O⋅⋅⋅H‐N), δ (N‐H⋅⋅⋅O = C) > δ (N‐D⋅⋅⋅O‐H/D)				

Except for CH_3_COOK, the loaded salts do not change the temporal sequence of solvation events in the PSBP films that is depicted as 1545 > 1620 > 1450 > 1655 cm^−1^. This sequence indicates that upon water absorption, the water shell surrounding the chemical groups responsible for the amide II band in the PSBP film builds up faster than the one surrounding the groups responsible for the amide I band. Later an H/D exchange follows. As a result, the hydrated water molecules break the intermolecular hydrogen bonds, leading to an expansion of the polymer chains. As listed in Table 1, the temporal sequence of solvation events is depicted as 1560 > 1620 > 1450 > 1545,1655 cm^−1^ for the CH_3_COO‾‐loaded PSBP film, as two autopeaks (*v* = 1580 and 1560 cm^−1^) are detected in the synchronous map (Figure S11a, Supporting Information). These are attributed to the hydration of CH_3_COO‾ as mentioned in analysis of the static FT‐IR spectra. Such finding implies that the hydration of CH_3_COO‾ is faster than of the amide groups, and makes the amide I band response prior to that of the amide II bands.

Significantly, as listed in Table 1, depending on the loaded anion species, the temporal sequence of solvation events differs among the various salt‐loaded PNIPMAM films. The same temporal sequences of 1605 > 1542 > 1665, 1500 cm^−1^ are found for Cl‾, I‾, and NO_3_‾‐loaded PNIPMAM films, indicating that the amide I related group responds faster than the amide II related one. A weak autopeak (*v* = 1460 cm^−1^), assigned to the amide II׳ band, is detected in CH_3_COO‾‐loaded and salt‐free synchronous maps, but a strong peak is found in the Br‾‐loaded synchronous map (Figure S12c, Supporting Information). Unexpectedly, the observed sequences indicate that response of the amide II׳ band is faster than the responses of the amide I and amide II bands, while the responses of the of amide I and II bands to hydration are faster than the breaking of intermolecular hydrogen bonds for CH_3_COO‾‐ and Br‾‐loaded samples but slower for the salt‐free sample.

These observations demonstrate that upon water absorption, the water shell surrounding the groups responsible for the amide I band in the PNIPMAM films builds up faster than the hydration shell around the ones producing the amide II band. As a result, the intermolecular hydrogen bonds break. When a preferential H/D exchange exists, the amide I and amide II bands show a similar response priority in their sequences. These differences in the PNIPMAM solvation sequences may be correlated to the findings for the salt series as determined by SR and FT‐IR, namely that the CH_3_COO‾‐ and Br‾ anions provoke a pronounced effect, and that the relative effect of Br‾ crosses that of CH_3_COO‾ at one point, while the Cl‾, I‾ and NO_3_‾ anions induce very similar effects.

For all DBC samples, the hydration events basically follow the sequence of 1605 > 1545 > 1465 cm^−1^, indicating that the amide I band response is faster than the one of the amide II band, while the amide II׳ band response is slowest. As for DBC films, in addition to the CH_3_COO‾ autopeak (*v* = 1580 cm^−1^) in Figure S13a (Supporting Information), an autopeak (*v* = 1400 cm^−1^) is seen and attributed to the hydration of NO_3_‾ (Figure S13e, Supporting Information). Besides, with CH_3_COO‾ present, the amide I band loses its priority, while with NO_3_‾ present, the amide II band reaches priority. These differences can explain why the salt effect of CH_3_COO‾ in the DBC film is less pronounced than in the PSBP and PNIPMAM films. Moreover, it can explain that NO_3_‾ has a priority to I‾ in the DBC salt series. We also find that the response of amide II׳ band takes precedence in PNIPMAM films but lags in PSBP films over the amide I and II bands and that the autopeaks assigned to amide II׳ band show up in Cl‾, I‾ NO_3_‾‐loaded PSBP film but not in PNIPMAM films. Taking these observations together, we conclude that the response of the PNIPMAM block is faster than the response of the PSBP block in the DBC films upon water hydration.

#### 2.2.5 Solvation Shell and the DBC Polymer Conformation

**Figure 9:**
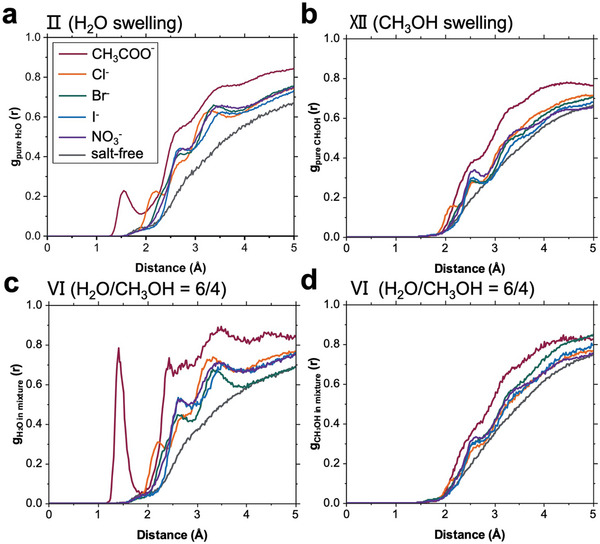
Calculated radial distribution function (RDF) profiles for the H_2_O shell in a) H_2_O and c) mixed H_2_O/CH_3_OH atmosphere, and for the CH_3_OH shell in b) CH_3_OH and d) mixed H_2_O/CH_3_OH atmosphere in the presence of different anions as indicated by the different colors.

To examine the salt effect on the accessibility of solvation shells near the hydrophilic groups, radial distribution function (RDF) profiles between solvation shells and DBC chains are calculated as shown in **Figure** **9**. We restrict the MD calculations to a single DBC chain, featuring 4 SBP repeating units and 6 NIPMAM repeating units exposed to pure H_2_O atmosphere (Figure S14, Supporting Information), pure CH_3_OH atmosphere (Figure S15, Supporting Information), and mixed H_2_O /CH_3_OH atmosphere (Figure S16, Supporting Information).

In a pure H_2_O vapor atmosphere (Figure 9a), the first H_2_O shell is observed at ≈1.5 Å and ≈2.2 Å respectively for CH_3_COO‾‐ and Cl‾‐loaded DBC chains, while the second H_2_O shell is observed for both at ≈2.7 Å. These values represent the hydrogen bond length, signifying the distance between hydrophilic groups and the hydration shell. A shorter distance indicates strong hydrogen bonding and a robust hydration shell surrounding the hydrophilic groups. In addition, the first H_2_O shell is observed at ≈2.7 Å for Br‾, I‾ and NO_3_‾‐loaded DBC chains after an RDF overlap at ≈2.5 Å. Yet before the overlap, a shift toward the right follows the tendency Br‾ > NO_3_‾ > I‾, as well as the intensity decrease in the RDF profiles. These findings indicate that the hydration water shells not close to the hydrophilic groups follow the anion sequence of CH_3_COO‾ < Cl‾ < Br‾ < NO_3_‾ < I‾. As shown in Figure S17 (Supporting Information), by integrating the corresponding relative intensity peak area, the accessibility of the water shell to the DBC chains obeys the anion order CH_3_COO‾ > Cl‾ > Br‾ > NO_3_‾ > I‾ of the salt series found in the SR, TOF‐NR, and FT‐IR data analysis.

In a pure CH_3_OH atmosphere (Figure S17, Supporting Information), the corresponding intensity peak area decreases, indicating that there are fewer methanol molecules than water molecules nearby the polymer chain. The observation that a first methanol shell is observed at ≈2.1 Å for Cl‾‐loaded and at ≈2.6 Å for the other salt‐loaded DBC chains, indicates that the presence of Cl‾ allows the methanol shell to be closer to the hydrophilic groups. Considering the integrated relative peak areas of the intensity in Figure S17 (Supporting Information), the salt series of CH_3_COO‾ > Cl‾ > Br‾ > NO_3_‾ > I‾ is still valid for the accessibility of the methanol shell to the salt‐loaded DBC chain.

Analogously, in a mixed H_2_O/CH_3_OH atmosphere, when comparing the integrated relative peak areas of the intensity in Figure S17 (Supporting Information), the accessibility of the water shell follows the order CH_3_COO‾ > Cl‾ > Br‾ > NO_3_‾ > I‾, while the methanol shell follows the slightly modified order CH_3_COO‾ > Cl‾ ≈ Br‾ ≈ NO_3_‾ ≈ I‾. Nevertheless, in each scenario, the loaded anions shift the formed solvation shell significantly closer to the DBC chains, especially in the cases of CH_3_COO‾ and Cl‾.

The conformation transitions of the salt‐free DBC chains in each case are illustrated by the snapshots from the MD simulations at 0 and 20 ps. As shown in Figure S18 (Supporting Information), the DBC chain undergoes a significant coil‐to‐globe transition from 0 to 20 ps, as the intermolecular hydrogen bonds are replaced by polymer‐water hydrogen bonds upon H_2_O hydration. In contrast, in the pure CH_3_OH atmosphere (Figure S19, Supporting Information), the DBC chain extends slightly due to the less cooperative methanol molecules. As both, water and methanol molecules, form hydrogen bonds with the hydrophilic groups in a mixed H_2_O/CH_3_OH atmosphere (Figure S20, Supporting Information), the DBC chains also expand slightly, but are surrounded by a built‐up methanol‐water shell.

## 3 Conclusion

The salt effects of five potassium salts, namely CH_3_COOK, KCl, KBr, KI, and KNO_3_, on the solvation behavior of thin films of the thermoresponsive zwitterionic polymer PSBP (UCST‐type), the polar non‐ionic one PNIPMAM (LCST‐type), and their diblock copolymer (DBC) PSBP‐*b*‐PNIPMAM (UCST‐*b*‐LCST type), are systematically studied via complementary experimental methods (SR, ToF‐NR, and FT‐IR) in pure water and mixed water/methanol vapor atmospheres. Salt‐free samples are studied as references. Complementary computational methods (2D FT‐IR analysis and MD simulations) explain the salt effects on the response sequence of solvation events and the accessibility of solvation shells.

For all combinations of polymers and salt, marked solvation effects are found. These vary however, for the different polymer types examined, and show also some polymer‐anion pair specificities. Moreover, the kinetics of the solvation events vary markedly for the various hydrophilic moieties involved, such as the sulfonate and the amide groups. For thin PSBP films, the efficiency of the salt effects on the solvation behavior of may be approximatively described by an inverted Hofmeister series as CH_3_COO‾ > Cl‾ > Br‾ > I‾ > NO_3_‾, with CH_3_COO‾ featuring the strongest and NO_3_‾showing the smallest effect, with respect on film thickness and solvent content. The order of salt effects found for the DBC films matches even better an inverted Hofmeister series, with CH_3_COO‾ > Cl‾ > Br‾ > NO_3_‾ > I‾. However, in the case of PNIPMAM thin films, such a description seems not appropriate, as the observed order in the salt series changes from Br‾ > CH_3_COO‾ > Cl‾ ≈ I‾ > NO_3_‾ to CH_3_COO‾ > Br‾ > Cl‾ > I‾ > NO_3_‾ when the *V_methanol_* exceeds 40%. Thus, in a 2D geometry hydrogel where the polymer is the majority component, the polymer density significantly influences polymer‐ion interactions, leading to solvation behavior (solvent absorption and polymer conformation) deviating from the traditional Hofmeister series observed in aqueous solutions, particularly in zwitterionic PSBP films with non‐specific ion interactions (ion‐polymer ionic groups).

The cosolvent effect depends on the response property (positive or negative) of the thin films. The reached equilibrated state is explained by the balance between cosolvent and salt effects. In pure water vapor, the salt effects are mainly manifested in the change of the water absorption, visualized in the temporal sequence of solvation events and the accessibility of the hydration water shells. In contrast, in the specific mixed water/methanol (6/4) vapor, they are mainly manifested in the regulation of the methanol absorption under retained moisture, visualizing in the examined water/methanol contents and a built‐up methanol‐water solvation shell. The presented findings provide potential applications and mechanism support (see Figure S21, Supporting Information) in the development of smart materials with tunable solvation properties, the design of more efficient separation membranes, and the optimization of drug delivery systems that leverage the unique behavior of thermoresponsive polymers in mixed solvent environments.

## 4 Experimental Section

### SR, ToF‐NR, and FT‐IR Spectroscopy

A SR measurement device (Filmetrics F20 Thin Film Measurement System, KLA, Milpitas, U.S.A.) was used to detect the film thickness. In situ, ToF‐NR measurements were conducted on the vertical sample plane reflectometer D17 by the time‐of‐flight (ToF) mode at Institut Laue‐Langevin (ILL, Grenoble, France).^[^ ^23^ ^]^ A Bruker Equinox 55 FT‐IR spectrometer was used to collect the FT‐IR spectra. A more detailed description is given in the Supporting Information.

### 2D FT‐IR Correlation Analysis

The 2D Shige software developed by Shigeaki Morita (Kwansei‐Gakuin University, Japan) was applied to generalize the 2D FT‐IR analysis, and the Origin software was further used to plot the resulting 2D synchronous and asynchronous maps combined with the corresponding FT‐IR spectra. Further analysis procedures based on Noda's rule^[^ ^22a,b^ ^]^ are described in the Supporting Information.

### MD Simulation

MD simulations were conducted using the Compass force field within the Forcite module, renowned for its accuracy in polymer systems, particularly those involving hydrogen bonding and other interactions critical to our study. A 1 fs time step was used to capture the rapid dynamics and hydrogen bonding interactions essential to the solvation behavior of the polymers. The system was equilibrated in the constant‐pressure ensemble (NPT) ensemble at a constant pressure of 0.1 MPa, maintained by a Berendsen barostat with a decay rate of 0.1 ps over 20 ps, while a Nose thermostat was used to control the temperature at 298 K, approximating physiological conditions.^[^ ^24^ ^]^ This setup ensures that the system's pressure remains constant, closely mimicking real‐world scenarios. Production runs were then carried out in the constant‐volume ensemble (NVT) ensemble for 200 ps to allow the system to reach equilibrium and to capture the steady‐state behavior of the polymers. The duration was chosen to provide ample time for the system to relax and for the polymer chains to fully explore their conformational space, enabling a statistically significant analysis of the results. Further simulation parameters are detailed in the Supporting Information.

### Statistical Analysis

The majority of experiments in this study were direct measurement outcomes or theoretical calculations based on raw data. In Figures 2, 4, and 5, the swelling ratio (*d/d_ini_*) represents the thickness (*d*) at time *t* divided by the initial thickness (*d_ini_*) before swelling. The *d_ini_* was determined from the average of hundreds of measurements taken in the half hour prior to swelling, with mean ± SD values detailed in the Supporting Information. In Figure 7, the normalization method for integrated peak areas, utilizing a three‐shoulder peak (labeled “b,” associated with the asymmetric stretching vibration of CH_2_/CH_3_ isopropyl groups) for normalization purposes, is detailed in the Supporting Information. All measurements were designed to characterize the in situ swelling behavior of the salt series‐driven system, including thickness, solvent content, and polymer‐solvent interactions, under specific mixed vapors, rather than to test for significant differences between various sample environments.

## Conflict of Interest

The authors declare no conflict of interest.

## Author Contributions

P.W. carried out the experimental measurements and wrote the original draft. T.Z., Y.‐J.C., and T.X. contributed to the MD simulations. J. R., S. A. W., L. F. H., and R.C. contributed to ToF‐NR measurements. L.P.K. and S.L. contributed to the data analysis. V.H. and A.L. contributed to polymer synthesis. All the authors discussed the results and contributed to the manuscript. P.M.‐B., A.L., and C.M.P. provided resources and funding. P.M.‐B. supervised the study.

## Supporting information



Supporting Information

## Data Availability

The data that support the findings of this study are available from the corresponding author upon reasonable request.

## References

[1] a) Y. Zhang , S. Furyk , D. E. Bergbreiter , P. S. Cremer , J. Am. Chem. Soc. 2005, 127, 14505; 16218647 10.1021/ja0546424

[2] a) F. Hofmeister , Arch. Exp. Path. Pharm. 1888, 24, 247;

[3] V. Hildebrand , A. Laschewsky , M. Päch , P. Müller‐Buschbaum , C. M. Papadakis , Polym. Chem. 2017, 8, 310.

[4] J. Niskanen , H. Tenhu , Polym. Chem. 2017, 8, 220.

[5] V. Aseyev , H. Tenhu , F. M. Winnik , Self Organized Nanostructures of Amphiphilic Block Copolymers II, Springer, Berlin Heidelberg 2011, 29.

[6] a) X. Liu , F. Cheng , H. Liu , Y. Chen , Soft Matter 2008, 4, 1991;

[7] a) Y. Zhang , S. Furyk , L. B. Sagle , Y. Cho , D. E. Bergbreiter , P. S. Cremer , J. Phys. Chem. C 2007, 111, 8916; 10.1021/jp0690603 PMC2553222 18820735

[8] C. Henschel , D. Schanzenbach , A. Laschewsky , C.‐H. Ko , C. M. Papadakis , P. Müller‐Buschbaum , Colloid Polym. Sci. 2023, 301, 703.

[9] C. Scherzinger , A. Schwarz , A. Bardow , K. Leonhard , W. Richtering , Curr. Opin. Colloid Interface Sci. 2014, 19, 84.

[10] M. J. Hore , B. Hammouda , Y. Li , H. Cheng , Macromolecules 2013, 46, 7894.

[11] J. Wang , N. Wang , B. Liu , J. Bai , P. Gong , G. Ru , J. Feng , Phys. Chem. Chem. Phys. 2017, 19, 30097. 29099128 10.1039/c7cp04384h

[12] S. C. Jung , S. Y. Oh , Y. ‐J. C. Bae , Polymer 2009, 50, 3370.

[13] S. Bharadwaj , B.‐J. Niebuur , K. Nothdurft , W. Richtering , N. van der Vegt , C. M. Papadakis , Soft Matter 2022, 18, 2884. 35311857 10.1039/d2sm00146b

[14] K. Pagonis , G. Bokias , Polym. Int. 2006, 55, 1254.

[15] a) Y. Matsuda , M. Kobayashi , M. Annaka , K. Ishihara , A. Takahara , Polym. J. 2008, 40, 479; 10.1021/la8005647 18627181

[16] a) L. Liu , T. Wang , C. Liu , K. Lin , G. Liu , G. Zhang , J. Phys. Chem. B 2013, 117, 10936; 23980605 10.1021/jp406215c

[17] T. López‐León , D. Bastos‐González , J. L. Ortega‐Vinuesa , A. Elaïssari , ChemPhysChem 2010, 11, 188. 20033975 10.1002/cphc.200900491

[18] V. Hildebrand , A. Laschewsky , E. Wischerhoff , Polym. Chem. 2016, 7, 731.

[19] P. Wang , C. Geiger , L. P. Kreuzer , T. Widmann , J. Reitenbach , S. Liang , R. Cubitt , C. Henschel , A. Laschewsky , C. M. Papadakis , P. Müller‐Buschbaum , Langmuir 2022, 38, 6934. 35609178 10.1021/acs.langmuir.2c00451

[20] E. O. Ningrum , S. Sakohara , T. Gotoh , N. Humaidah , Polymer 2020, 186, 122013.

[21] P. Wang , C. Geiger , J. Reitenbach , A. Vagias , L. P. Kreuzer , S. Liang , R. Cubitt , V. Hildebrand , A. Laschewsky , C. M. Papadakis , P. Müller‐Buschbaum , Macromolecules 2023, 56, 4087.

[22] a) I. Noda , A. E. Dowrey , C. Marcott , G. M. Story , Y. Ozaki , Appl. Spectrosc. 2000, 54, 236A;

[23] P. Müller‐Buschbaum , R. Cubitt , I. F. Huber , M. Le Dû , J. Reitenbach , P. Wang , S. A. Wegenr , Institut Laue‐Langevin (ILL) 2023 10.5291/ILL-DATA.9-11-2093.

[24] a) T. Zheng , J. Xiong , B. Zhu , X. Shi , Y.‐J. Cheng , H. Zhao , Y. Xia , J. Mater. Chem. A 2021, 9, 9307; 10.1021/acsami.1c15690 34797642

