# Supplementary material for: Solvation Dynamics of Thermoresponsive Polymer Films: The Influence of Salt Series in Water and Mixed Water/Methanol Atmosphere
Source: Adv Sci (Weinh). 2024 Dec 31;12(8):2408073. doi: 10.1002/advs.202408073 (PMC11848558; doi:10.1002/advs.202408073)
Supplement: Supplementary file 1 — Supporting Information [file ADVS-12-2408073-s001.pdf]

ADVANCED SCIENCE
Open Access

Supporting Information

for *Adv. Sci.*, DOI 10.1002/advs.202408073

Solvation Dynamics of Thermoresponsive Polymer Films: The Influence of Salt Series in Water and Mixed Water/Methanol Atmosphere

*Peixi Wang, Tianle Zheng, Julija Reitenbach, Simon A. Wegener, Linus F. Huber, Lucas P. Kreuzer, Suzhe Liang, Robert Cubitt, Ya-Jun Cheng, Tonghui Xu, Viet Hildebrand, André Laschewsky, Christine M. Papadakis and Peter Müller-Buschbaum**

Supplementary Information

Solvation Dynamics of Thermoresponsive Polymer Films: The Influence of Salt Series in Water and Mixed Water/Methanol Atmosphere

Peixi Wang[1], Tianle Zheng[1], Julija Reitenbach[1], Simon A. Wegener[1], Linus F. Huber[1], Lucas P. Kreuzer[2], Suzhe Liang[1], Robert Cubitt[3], Ya-Jun Cheng[4], Tonghui Xu[5], Viet Hildebrand[6], André Laschewsky[6,7], Christine M. Papadakis[8], Peter Müller-Buschbaum[1,]*

[1]Technical University of Munich, TUM School of Natural Sciences, Department of Physics, Chair for Functional Materials, James-Franck-Str. 1, 85748 Garching, Germany

[2]Heinz Maier-Leibnitz Zentrum (MLZ), Technical University of Munich, Lichtenbergstr. 1, 85748 Garching, Germany

[3]Institut-Laue-Langevin, 6 rue Jules Horowitz, 38000 Grenoble, France

[4]Ningbo Institute of Materials Technology & Engineering, Chinese Academy of Sciences, 1219 Zhongguan West Rd, Ningbo, 315201, Zhejiang Province, P.R. China

[5]Department of Chemistry, College of Sciences. Shanghai University, 200444, Shanghai, P. R. China

[6]Institut für Chemie, Universität Potsdam, Karl-Liebknecht-Str. 24-25, 14476 Potsdam-Golm, Germany

[7]Fraunhofer Institut für Angewandte Polymerforschung, Geiselbergstr. 69, 14476 Potsdam-Golm, Germany

[8]Technical University of Munich, TUM School of Natural Sciences, Department of Physics, Soft Matter Physics Group, James-Franck-Str. 1, 85748 Garching, Germany

* Corresponding Author

Emails: muellerb@ph.tum.de

Supplementary Methods

Materials.

The synthesis of polymers PSBP, PNIPMAM and PSBP-*b*-PNIPMAM via reversible addition-fragmentation chain transfer (RAFT) polymerization was described previously.[1] The water (H_2O) was first deionized and additionally treated by a Millipore Milli-Q Plus water purification system. 2,2,2-trifluoroethanol (TFE, 99.8%, Roth), potassium salts CH_3COOK, KCl, KBr, KI, and KNO_3 ($\geq$ 99.0%, Sigma-Aldrich), deuterated water (D_2O, 99.95 %, Deutero), and deuterated methanol (methanol-d_3, CD_3OH, 99.95 %, Deutero) were used as received.

Sample preparation.

Polymer thin films were prepared by spin-coating to have a thickness of around 80 - 100 nm for SR measurements (initial thickness $d_{ini} \approx$ 91 – 98 ± 2 nm for PSBP, 83 - 91 ± 2 nm for PNIPMAM, and 98 - 101 ± 2 nm for PSBP-*b*-PNIPMAM), and with thicknesses of around 30 - 35 nm ± 2 for ToF-NR measurements. The polymer films with a thickness of 4 - 6 ± 0.4 µm for FT-IR measurements were prepared by the drop-casting method.

All polymers were dissolved in 2,2,2-trifluoroethanol (TFE) with a concentration of $c_{polymer}$ = 22.22 g/L. The potassium salts CH_3COOK, KCl, KBr, KI and KNO_3 were dissolved in deionized water with a concentration of c_{salts} = 100 mM. The various salt-loaded (or salt-free) polymer solutions used for the spin-coating and drop-casting process were prepared by mixing 9 parts of polymer stock solution with 1 part of salt solution (or of deionized water for the salt-free control samples).

The Si wafers were cut into a size of 20 mm × 20 mm for SR measurements, 70 mm × 70 mm for ToF-NR measurements and 10 mm × 10 mm for FT-IR measurements. The wafers used for spin-coating were cleaned by an acid bath (54 mL deionized H_2O, 84 mL H_2O_2, 198 mL H_2SO_4) at 80 °C for 20 min first, and then rinsed with deionized water three times to remove the retained acid. After drying under an N_2 stream, the wafers were saved in a plastic sample box. Before spin-coating, an oxygen plasma (2000 W for 10 min) was used to clean the surface. After coating with the prepared solutions (300 µL for SR and 2 mL for ToF-NR), the polymer films were prepared by spin-coating (2000 rpm and 90 s for SR and 3500 rpm and 120 s for ToF-NR

measurements). After that, all samples were annealed under solvent vapor for 15 min at room temperature. The wafers used in the drop-casting method were just cleaned by an oxygen plasma (2000 W for 10 min) before being used. For a good FT-IR signal-to-noise ratio, 60 µL of prepared solutions was drop-casted on the Si wafer and then transferred to a desiccator to heal naturally.

Sample environment.

Applied chambers: A 3D-printed aluminum chamber was used for SR and ToF-NR measurements, and a custom-made copper chamber was used for FT-IR measurements.[2] A thermal bath (JULABO FP50 HL) was connected to both chambers to achieve a constant temperature of 18 °C, and an SHT31 humidity and temperature sensor (Sensirion AG, Staefa, Switzerland) was plugged or fixed into the chambers to monitor the real-time temperature and the humidity change. In our current study, we opted to maintain a constant temperature to concentrate on the impact of salts on this behavior. This approach helped to isolate the effects of salts, providing a more focused analysis of their influence on co-nonsolvency without the confounding factors associated with temperature fluctuations. Besides, the 3D-printed aluminum chamber is equipped with a CaF_2 window on tap, and the customized copper chamber is equipped with two highly transmissive ZnS windows. As illustrated in a previous study,[3] the custom-made gas flow system with three channels was used to generate the N_2 flow or saturated vapors.

Applied vapor protocols (Figure S1): (1) For the stepwise vapor exchange protocol (Figure S1a), after drying under an N_2 stream, the samples were exposed to a saturated H_2O vapor (flow rate = 1 L/min, set as 100 %). Then, the saturated H_2O vapor was stepwise replaced by a methanol vapor in 10 vol% increments until a saturated methanol vapor was generated. Each step took 2 h and 2-hour intervals sufficed for our measurements, which showed that the film thickness generally stabilized, particularly in areas with abundant H_2O or methanol. We deduced that this duration was adequate for polymer chains to swell, especially with sufficient vapor flow from solvents. However, we recognized that this interval may not capture the swelling process at the macromolecular level during solvent exchange phases. Nonetheless, we asserted that it did not substantially affect the overall trend of thickness reduction in response

to varying vapor compositions. (2) For the mixed water/methanol vapor switching protocol (Figure S1b), all samples were dried under a N_2 flow before exposure under a saturated water vapor (H_2O for SR, or D_2O for ToF-NR and FT-IR). Once a plateau upon water hydration was reached, the saturated water vapor was switched to a saturated mixed vapor (H_2O/methanol for SR, or D_2O/methanol-d_3 = 6/4 for ToF-NR and FT-IR). Then, the reached equilibrium ended the measurement protocol; (3) For the methanol vapor switching protocol (Figure S1c) for SR measurements, the dried samples were directly exposed to a saturated methanol vapor.

Spectral reflectance (SR).

As described above, a CaF_2 window existed on the top of the 3D aluminum chamber mentioned above, which allowed the white light to pass through. The white-light source was fixed at 10 cm above the thin films.

By fitting a multilayer model, the resulting optical film thickness d_{opt} and refractive index n were acquired by the FILMeasure software (KLA, Milpitas, U.S.A.). Thus, the real geometric thickness d_{real} was determined with the Equation S1.

$$\mathrm{d_{opt}} = \mathrm{d_{real}}\ \mathrm{n} \tag{S1}$$

Here, the d_{opt} was obtained by fits to the interference patterns, and the refractive index n of the d_{real} was obtained by the Lorentz-Lorenz effective medium approximation as Equation S2.

$$\frac{\mathrm{n_{real}^2-1}}{\mathrm{n_{real}^2+2}} = \frac{\mathrm{d_{ini}}}{\mathrm{d_{real}}}\frac{\mathrm{n_{polymer}^2-1}}{\mathrm{n_{polymer}^2+2}} + \frac{\mathrm{d_{real}-d_{ini}}}{\mathrm{d_{real}}}\frac{\mathrm{n_{solvent}^2-1}}{\mathrm{n_{solvent}^2+2}} \tag{S2}$$

where, n_{DBC}, $n_{solvent}$ represent the refractive index of polymer and solvent, respectively. Here, $n_{polymer}$ was set to n = 1.5 and $n_{solvent}$ ($n_{H2O} = n_{D2O} = n_{methanol\text{-}d3}$) was set to n = 1.33. Since the volume fractions were calculated via d_{ini}/d_{real}, and $(d_{real} - d_{ini})/d_{real}$, respectively. Thereby, the real geometric thickness d_{real} was determined by implementing Equation S1 in Equation S3:

$$\frac{d_{opt}^2-d_{real}^2}{d_{opt}^2+2d_{real}^2} = \frac{d_{ini}}{d_{real}}\frac{n_{polymer}^2-1}{n_{polymer}^2+2} + \frac{d_{real}-d_{ini}}{d_{real}}\frac{n_{solvent}^2-1}{n_{solvent}^2+2} \tag{S3}$$

This decoupling method was applied to determine the real geometric thickness from all SR data.

Time-of-flight neutron reflectometry (ToF-NR).

In situ ToF-NR measurements were performed in two working modes (static and kinetic) with a broad range of incident neutron wavelengths (λ = 2 – 27 Å, $\Delta\lambda/\lambda$ = 10 %). The reflected

neutrons were recorded as a function of wavelength λ and exit angle α_f at a sample-detector distance of 3.1 m. Three static NR measurements for each sample were conducted after the N_2 drying, the D_2O swelling and the vapor switching processes. Two fixed incident angles of α_i = 0.5° (10 min) and α_i = 2.5° (35 min) were applied to get a wide range of scattering vectors from q_z = 0.01 to 0.25 Å^{-1}. For the kinetic NR measurements, the incident angle was first set to α_i = 1.0° for a good time resolution and then moved to α_i = 1.5°, to protect the detector from oversaturation. *In situ* ToF-NR measurements were performed for 3 h for the D_2O swelling process and performed for 4.5 h for the vapor switching process.

The Igor Pro software (WaveMetrics, Portland, U.S.A.) with the Motofit package (Andrew Nelson, Australian NuclearScience and Technology Organiza) was used for NR data analysis.[4] The resulting film thickness and the corresponding SLD profiles were obtained. Furthermore, the absorbed D_2O and methanol-d_3 content was calculated from the acquired SLD profile following the previous approach below.[2b, 3, 5]

The overall solvent content is calculated by thickness and SLD as

$$\Phi_{solvent}(t) = \frac{SLD_{meas}(t) - SLD_{salt-loaded\ DBC}}{SLD_{solvent} - SLD_{salt-loaded\ DBC}} \quad \text{(S4)}$$

Here, $SLD_{meas}(t)$ represents the measured SLD at time t from a three-layer model, including a substrate-polymer layer, a bulk polymer layer and a polymer-air layer, and $SLD_{salt\text{-}loaded\ DBC}$ is the calculated salt-loaded DBC SLD ($SLD_{Br^-\text{-}loaded\ DBC}$ = 0.806 × 10^{-6}Å^{-2}, and $SLD_{NO3^-\text{-}loaded\ DBC}$ = 0.871 × 10^{-6}Å^{-2}), as listed in Table S2. Since we presumed that the density is 1.06 g/cm^3 and 1.1 g/cm^3 for PSBP and PNIPMAM blocks respectively, the calculated density of DBC is 1.06 g/cm^3.[6] Thus, the D_2O content during the D_2O swelling processes was determined by Equation S4 with temporal evolution of the bulk SLD in Figure 4b. Upon the vapor switching process, two solvent contents must be considered. Thus, the molar fraction of methanol-d_3 $x_{methanol\text{-}d3}(t)$ at time t is introduced as

$$SLD_{solvent}(t) = (1 - x_{methanol-d3}(t))\ SLD_{D2O} + x_{methanol-d3}(t)\ SLD_{methanol-d3} \quad \text{(S5)}$$

We assume that the solvent content was related to the film thickness change via a volumetric conversion factor γ as

$$\gamma = \frac{\frac{SLD_{meas}(t)\ -\ SLD_{salt-loaded\ DBC}}{SLD_{solvent}(t)\ -\ SLD_{salt-loaded\ DBC}}}{\frac{d_{ini}}{d_{meas(t)}}\ -\ 1} \tag{S6}$$

During vapor switching processes, the value of γ was approximated as 0.5 for Br^--loaded DBC film and 0.48 for NO_3^--loaded DBC film. After implementing Equation S6 in Equation S5, the methanol-d3 molar fraction $x_{\text{methanol-d3}}(t)$ at time t was calculated as below and the temporal evolution of the determined methanol-d3 molar faction $x_{\text{methanol-d3}}$ was plotted in Figure S4a.

$$x_{methanol-d3}(t) = \frac{\left(\frac{SLD_{meas}(t)\ -\ SLD_{salt-loaded\ DBC}}{\gamma\left(\frac{d_{ini}}{d_{meas(t)}}\ -\ 1\right)}\right) + SLD_{salt-loaded\ DBC} - SLD_{D2O}}{SLD_{methanol-d3}\ -\ SLD_{D2O}} \tag{S7}$$

Thus, upon vapor switching processes, the overall absorbed solvent content was calculated by Equation S6 and plotted in Figure S4b. As a result, the absorbed D_2O content and the methanol-d3 content upon vapor switching were calculated and plotted in Figure 4c and d respectively.

Fourier-transform infrared (FT-IR) spectroscopy.

The employed Bruker Equinox 55 FT-IR spectrometer was equipped with a KBr beam splitter and a DTGS detector. FT-IR spectra were collected in transmission mode, recorded from v = 4000 - 600 cm^{-1} with a spectral resolution of 2 cm^{-1}. This configuration allowed recording spectra *in situ* (time resolution of 1.5 min for 50 scans).

For further normalization of FT-IR spectra, the peak with three shoulders (indicated as "b", $v \approx$ 2950 cm^{-1}), mainly assigned to CH_2/CH_3 isopropyl asymmetric stretching vibration excited in the polymer backbone and side chains and known for its hydrophobicity in aqueous, showed almost imperceptible changes neither upon water hydration nor vapor switching. Thus, this three-shoulder peak was used for the normalization of the integrated peak areas. Besides, since its hydrophobicity affects slightly to phase transition behavior at both kinetics, only the salt effect on hydrophilic surroundings is discussed in the FT-IR part, as well as in the 2D FT-IR part.

The static FT-IR spectra are shown in Figure S11. The spectra were recorded as the last spectrum upon N_2 drying, D_2O swelling and vapor switching. The FT-IR peaks derived from Cl^-, Br^-, I^- are not marked in figures, since those peaks present at lower wavenumbers ($v_{(Cl^-, Br^-,}$

$_{I^{-}}$) ≈ 800-600 cm^{-1}).

Two-dimensional FT-IR (2D FT-IR) correlation analysis.

Only the first 30 FT-IR spectra during D_2O swelling were used for the 2D correlation analysis to determine the temporal sequence of the fluctuation in spectral intensity of amide bands. The resulting synchronous and asynchronous maps were marked as Φ and Ψ respectively, and two independent wavenumber axes were set as v_1 and v_2 (v_1 refers to the y-axis and v_2 refers to the x-axis). The positive correlation and negative correlation were colored in red and blue respectively.

According to Noda's rule[7], if the cross-peak shows the same coloration in both synchronous and asynchronous maps ($\Phi(v_1, v_2) > 0$, $\Psi(v_1, v_2) > 0$ or $\Phi(v_1, v_2) < 0$, $\Psi(v_1, v_2) < 0$), the event at v_1 will vary prior to that at v_2. Reversely, if $\Phi(v_1, v_2) > 0$, $\Psi(v_1, v_2) < 0$ or $\Phi(v_1, v_2) < 0$, $\Psi(v_1, v_2) > 0$, the event at v_1 will vary after that at v_2.

For example, in Figure 8a, in off-diagonal region ($v_1 > v_2$) of both synchronous and asynchronous maps, the cross-peaks centered at the (1620, 1655), (1545, 1620), (1450, 1655) cm^{-1} are negative and the cross-peak centered at the (1545, 1655) cm^{-1} is positive, While, the correlation of cross-peaks centered at the (1450, 1545), (1450, 1620) cm^{-1} are exactly opposite in both maps. Thus, according to Noda's rule, the concluded sequence of solvation events for the Cl^--loaded PSBP sample is depicted as 1545 > 1620 > 1450 > 1655 cm^{-1}. Similarly, in Figure 8b, the cross-peaks centered at the (1605, 1665), (1542, 1665) cm^{-1} are both negative while the cross-peaks centered at the (1500, 1605), (1500, 1542), (1542, 1605) cm^{-1} are opposite in 2D maps. Hence, the concluded sequence of solvation events for the Cl^--loaded PNIPMAM sample is depicted as 1605 > 1542 > 1665, 1500 cm^{-1}. Concerning the observations in the Cl^--loaded DBC sample, the cross-peaks centered at the (1605,1667), (1545, 1667) cm^{-1} are both negative while the cross-peaks centered at the (1465, 1667), (1465, 1605), (1465, 1545), (1465, 1505) (1505, 1605), (1545, 1605) cm^{-1} are opposite in two maps, the concluded sequence of solvation events is depicted as 1605 > 1545 > 1667, 1505 > 1465 cm^{-1}.

Molecular dynamics (MD) simulation.

Quantum chemical calculations were performed using the Dmol3 module with B3LYP

functional, custom Grimme DFT-D parameters, and DNP 4.4 basis set in the Material Studio package.[8] All components (CH_3COOK, KCl, KBr, KI, KNO_3, H_2O, CH_3OH, PSBP (4-unit)-*b*-PNIPMAM (6-unit)) utilized ESP charges after ultra-fine optimization and were simulated in a solvent environment using the conductor-like screening model (COSMO). The k-points were set to Gamma (1 × 1 × 1), and convergence tolerance was set at 1.0×10^{-5} Ha for energy and 2.0×10^{-3} Ha/Å $^{-1}$ for maximum force and maximum displacement of 5.0×10^{-3} Å. Several solution models were constructed to simulate the salt effect of the five potassium salts on solvation shells near the studied DBC chains in a pure H_2O (Figure S15), pure CH_3OH (Figure S16), and mixed H_2O/CH_3OH (6:4 v./v., Figure S17) atmosphere. Each system was designed to maintain a mole ratio of 112: 5: 944 for potassium salts, DBC chains, and solvent molecules, respectively.

Table S1 Summary of calculated theoretical SLD values, and the anionic radii.

$SLD_{neutron} \times 10^{-6}$ (Å^{-2})					$r_{ion} \times 10^{-3}$ (Å)	
H_2O	-0.559	DBC	0.772		CH_3COO^-	400
D_2O	6.393	KBr	1.456		Cl^-	181
CD_3OH	4.279	KNO_3	3.260		Br^-	196
Si	2.07	DBC with 10 mM KBr	0.806		I^-	220
SiO_2	3.47	DBC with 10 mM KNO_3	0.871		NO_3^-	177
					K^+	138

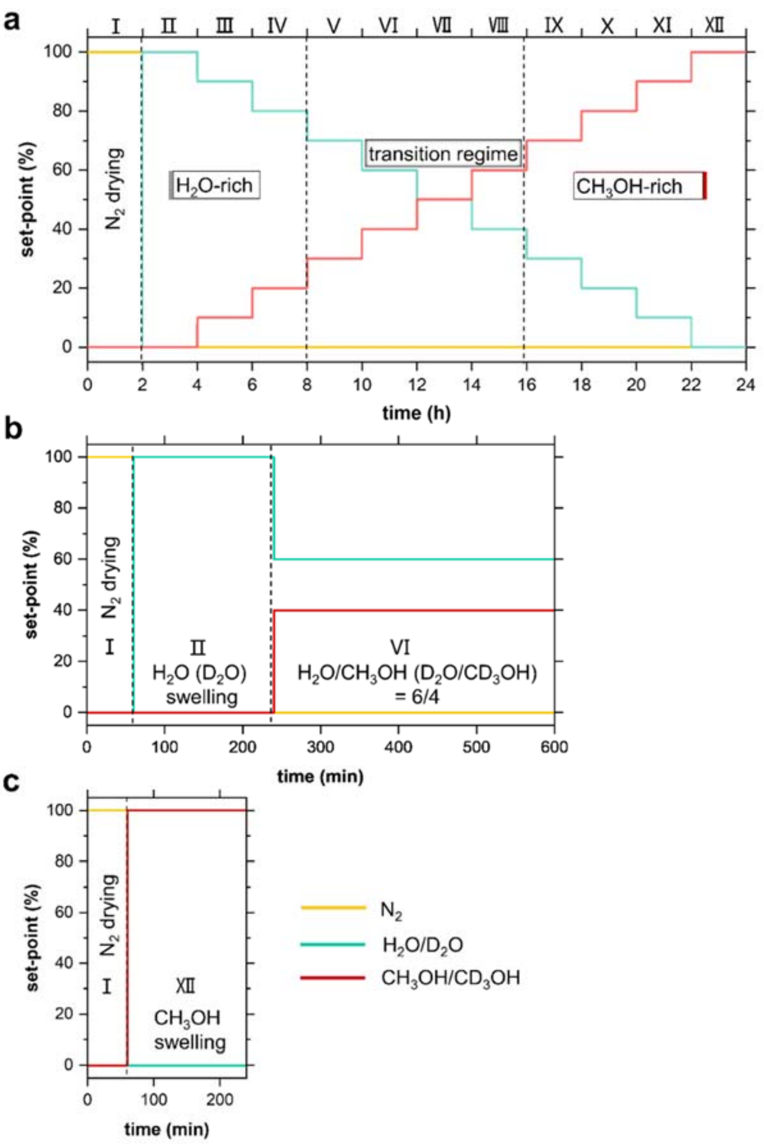

Figure S1. Applied vapor exchange and switching protocols for the corresponding *in situ* SR, ToF-NR, and FT-IR measurements. The flow rate is set as a set-point (100 % represents the flow rate = 1 L/min) and plotted in yellow, green, and red for N_2, H_2O/D_2O, and CH_3OH/CD_3OH, respectively.

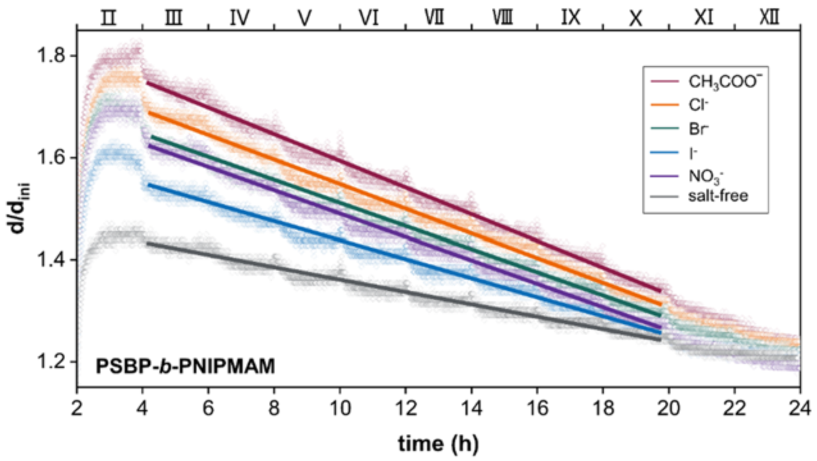

Figure S2. Enlarged evolution of the swelling ratio (d/d_{ini}) obtained from SR measurements as a function of time for PSBP-*b*-PNIPMAM thin films following a stepwise exchange of the surrounding vapor atmosphere. The solid lines indicate the near-linear collapses.

Table S2. Film thickness d, swelling ratio d/d_{ini}, bulk SLD, and D_2O and CD_3OH content of both Br^-- and NO_3^--loaded PSBP-*b*-PNIPMAM thin films at the three static stages.

Br^-	N_2 drying Ⅰ	D_2O swelling Ⅱ	vapor switching Ⅵ
d (nm)	34 ± 3	94 ± 3	60 ± 3
d/d_{ini}	1.0	2.76 ± 0.03	1.76 ± 0.05
SLD ×10^{-6} (Å^{-2})	0.78 ± 0.02	5.75 ± 0.05	1.85 ± 0.03
D_2O content ϕ_{D_2O} (vol %)	—	78 ± 2	15 ± 4
CD_3OH content ϕ_{CD3OH} (vol %)	—	—	20 ± 4
NO_3^-	N_2 drying Ⅰ	D_2O swelling Ⅲ	vapor switching Ⅵ
d (nm)	31 ± 2	82 ± 4	53 ± 2
d/d_{ini}	1.0	2.64 ± 0.04	1.70 ± 0.02
SLD ×10^{-6} (Å^{-2})	0.84 ± 0.02	4.85 ± 0.07	1.58 ± 0.06
D_2O content ϕ_{D_2O} (vol %)	—	67 ± 3	9 ± 1
CD_3OH content ϕ_{CD3OH} (vol %)	—	—	13 ± 2

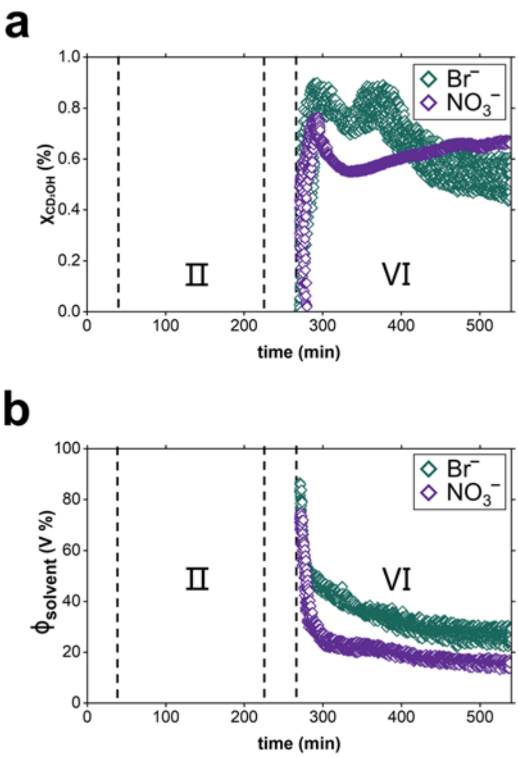

Figure S3. Evolution of the a) determined CD_3OH molar faction x_{CD_3OH}, b) the overall solvent content obtained from ToF-NR data as a function of time for Br^-- (green) and NO_3^--loaded (purple) PSBP-*b*-PNIPMAM thin films with the stages: D_2O swelling (II) and D_2O/CD_3OH = 6/4 (VI).

Table S3. Detailed IR band assignments of the three types of polymers.

detailed band assignments of PSBP, PNIMAM, PSBP-*b*-PNIPMAM in thin film geometry according to FTIR spectrum						
	**PSBP**		**PNIPMAM**		**PSBP-*b*-PNIPMAM**	
	wavenumber/cm^{-1}	assignment	wavenumber/cm^{-1}	assignment	wavenumber/cm^{-1}	assignment
**a**	3658 – 3145<br>CD_3OH<br>H_2O	$\nu_{as}(CD_3O\text{-}H)$	3600 – 3135<br>CD_3OH<br>H_2O	$\nu_{as}(CD_3O\text{-}H)$	3640 – 3155<br>CD_3OH<br>H_2O	$\nu_{as}(CD_3O\text{-}H)$
		$\nu_{as}(O\text{-}H_2)$		$\nu_{as}(O\text{-}H_2)$		$\nu_{as}(O\text{-}H_2)$
**b**	3090 - 2790	$\nu_{as}(N\text{-}H)$	3045 - 2850	$\nu_{as}(N\text{-}H)$	3060 - 2850	$\nu_{as}(N\text{-}H)$
		$\nu_{as}(C\text{-}H_3)$		$\nu_{as}(C\text{-}H_3)$		$\nu_{as}(C\text{-}H_3)$
		$\nu_{as}(C\text{-}H_2)$		$\nu_{as}(C\text{-}H_2)$		$\nu_{as}(C\text{-}H_2)$
		$\nu_{as}(C–H)$		$\nu\,(C–H)$		$\nu\,(C–H)$
		$\nu_{sym}(C\text{-}H_3)$		$\nu_{sym}(C\text{-}H_3)$		$\nu_{sym}(C\text{-}H_3)$
		$\nu_{sym}(C\text{-}H_2)$		$\nu_{sym}(C\text{-}H_2)$		$\nu_{sym}(C\text{-}H_2)$
**c**	2755 – 2235<br>D_2O	$\nu_{as}(O\text{-}D_2)$	2750 – 2185<br>D_2O	$\nu_{as}(O\text{-}D_2)$	2750 – 2185<br>D_2O	$\nu_{as}(O\text{-}D_2)$
**d**	2100 – 2020<br>CD_3OH	$\nu_{as}(C\text{-}D_3OH)$	2100 – 2020<br>CD_3OH	$\nu_{as}(C\text{-}D_3OH)$	2120 – 2020<br>CD_3OH	$\nu_{as}(C\text{-}D_3OH)$
**e**	1750 – 1575<br>*amide I*	$\nu_{as}(C{=}O\cdots H\text{-}N)$	1755 – 1570<br>*amide I*	$\nu_{as}(C{=}O\cdots H\text{-}N)$	1700 – 1575<br>*amide I*	$\nu_{as}(C{=}O\cdots D\text{-}N)$
		$\nu_{as}(C{=}O\cdots D\text{-}O\text{-}D)$		$\nu_{as}(C{=}O\cdots D\text{-}O\text{-}D)$		$\nu_{as}(C{=}O\cdots D\text{-}O\text{-}D)$
		$\nu_{as}(C{=}O\cdots H\text{-}O\text{-}D_3C)$		$\nu_{as}(C{=}O\cdots H\text{-}O\text{-}D_3C)$		$\nu_{as}(C{=}O\cdots H\text{-}O\text{-}D_3C)$
**f**	1575 – 1510<br>*amide II*	$\delta\,(N\text{-}H\cdots O{=}C)$	1570 – 1495<br>*amide II*	$\delta\,(N\text{-}H\cdots O{=}C)$	1575 – 1505<br>*amide II*	$\delta\,(N\text{-}H\cdots O{=}C)$
		$\delta\,(N\text{-}H\cdots O\text{-}D_2)$		$\delta\,(N\text{-}H\cdots O\text{-}D_2)$		$\delta\,(N\text{-}H\cdots O\text{-}D_2)$
		$\delta\,(N\text{-}H\cdots H\text{-}O\text{-}D_3C)$		$\delta\,(N\text{-}H\cdots H\text{-}O\text{-}D_3C)$		$\delta\,(N\text{-}H\cdots H\text{-}O\text{-}D_3C)$
**g**	1510 – 1340<br>*amide II'*	$\delta\,(N\text{-}D\cdots O\text{-}H/D)$	1495 – 1410<br>*amide II'*	$\delta\,(N\text{-}D\cdots O\text{-}H/D)$	1505 – 1405<br>*amide II'*	$\delta\,(N\text{-}D\cdots O\text{-}H/D)$
		$\delta_{as}(C–H_3)$		$\delta_{as}(C–H_3)$		$\delta_{as}(C–H_3)$
		$\delta_{as}(C–H_2)$		$\delta_{as}(C–H_2)$		$\delta_{as}(C–H_2)$
**h**	1410 - 1350	$\delta_{sym}(C–H_3)$	1410 - 1335	$\delta_{sym}(C–H_3)$	1405 - 1335	$\delta_{sym}(C–H_3)$
		$\delta_{sym}(C–H_2)$		$\delta_{sym}(C–H_2)$		$\delta_{sym}(C–H_2)$
**i**	1305 – 1265<br>amide III	——	1295 – 1235<br>*amide III*	——	1305 – 1265<br>*amide III*	——
**j**	1265 - 1080	CH_3 skeletal	1235 - 1005	CH_3 skeletal	1265 - 1100	CH_3 skeletal
		CH_2 skeletal		CH_2 skeletal		CH_2 skeletal
		CH_3 rocking		CH_3 rocking		CH_3 rocking
		CH_2 rocking		CH_2 rocking		CH_2 rocking
k	1080 - 1010	$\delta\,(\text{-}SO_2\text{-}O\text{-})$	——	——	1070 - 1010	$\delta\,(\text{-}SO_2\text{-}O\text{-})$

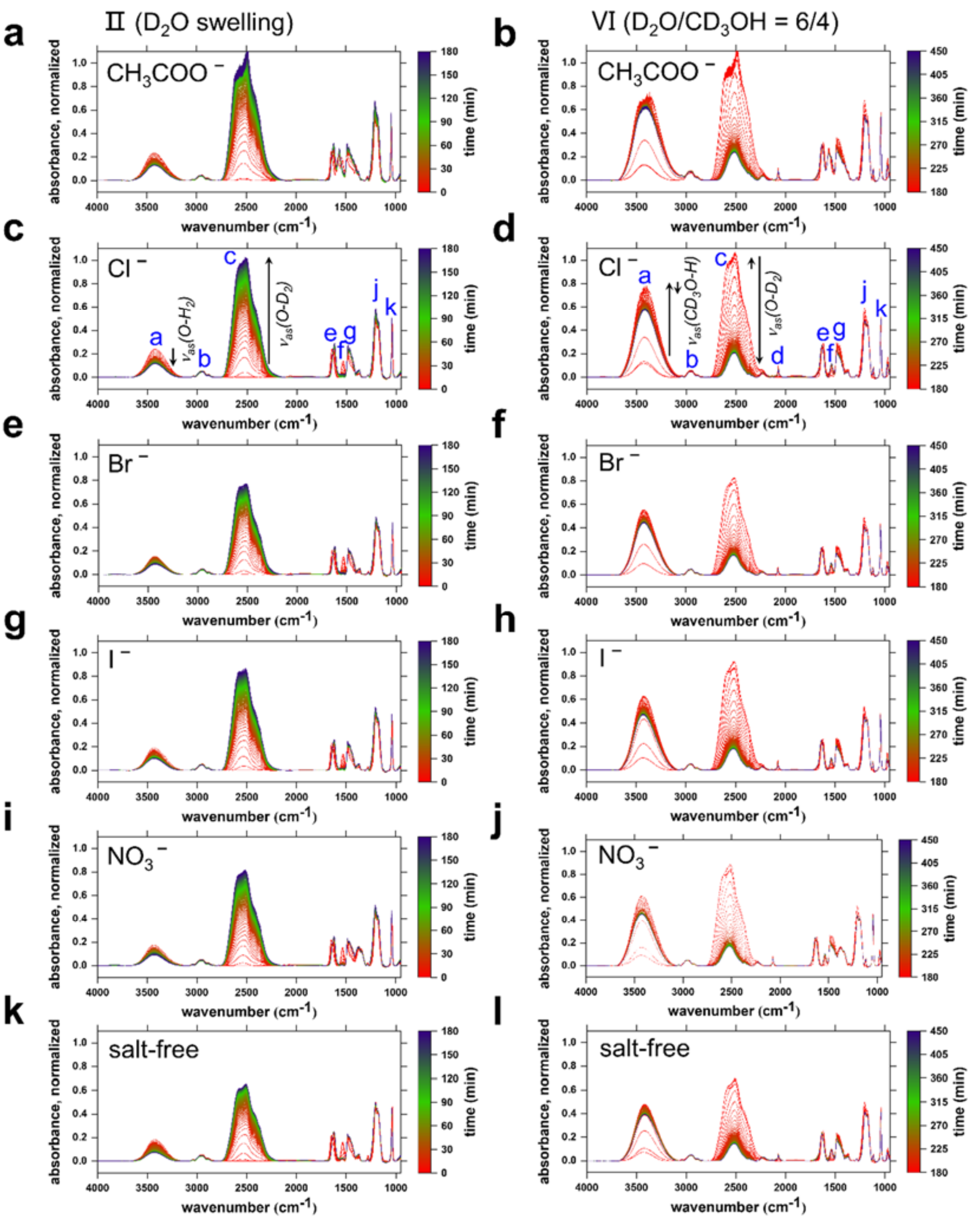

Figure S4. FT-IR spectra of a, b) CH_3COO^-, c, b) Cl^-, e, f) Br^-, g, h) I^-, i, j) NO_3^--loaded and k, l) salt-free PSBP thin films upon a, c, e, g, I, k) D_2O swelling (stage II) and b, d, f, h, j, l) subsequent vapor switching (stage VI). The characteristic bands listed in Table S3 are indicated by blue lower-case letters in c) and d), and the black arrows highlight the change of corresponding characterized signals.

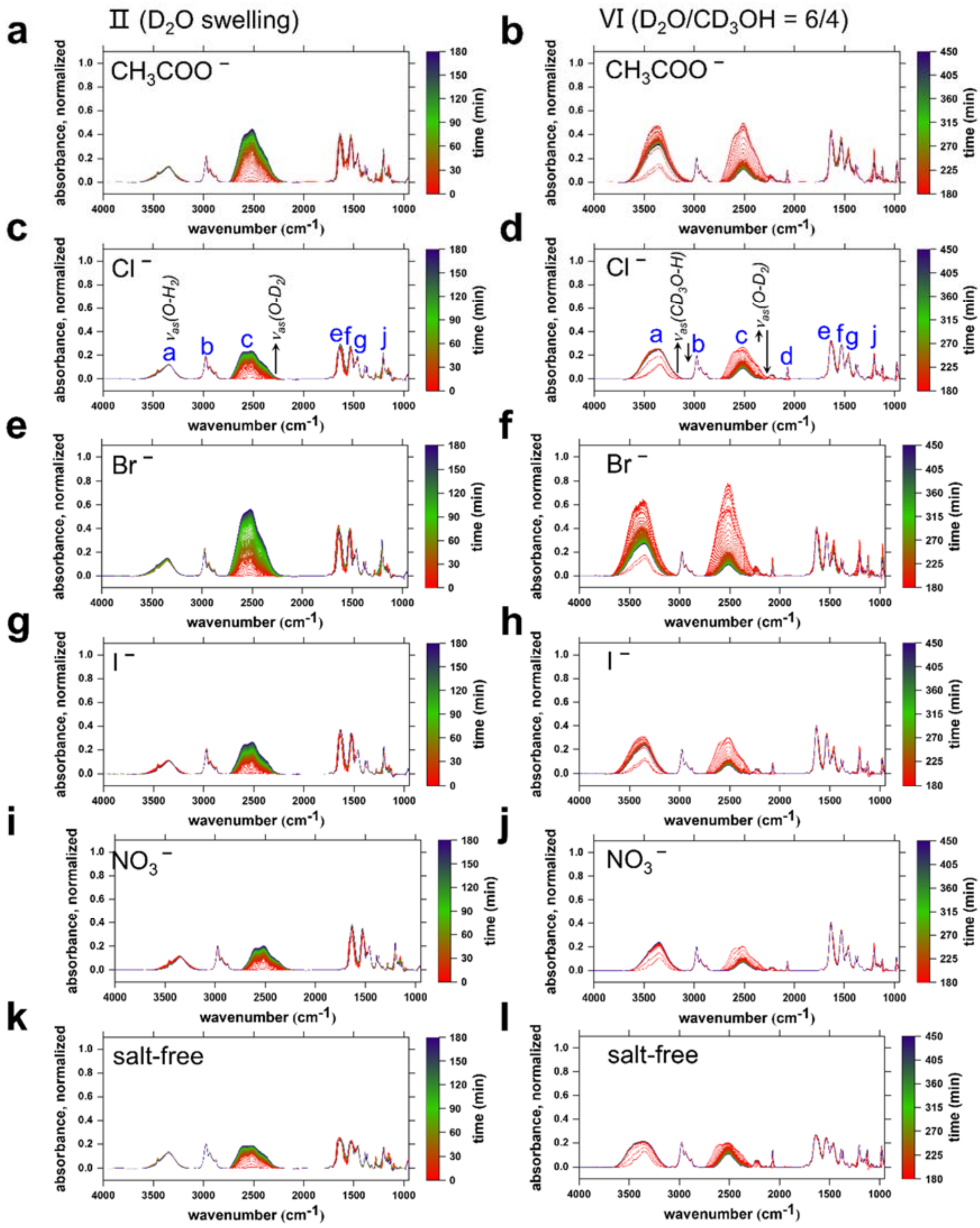

Figure S5. FT-IR spectra of a, b) CH_3COO^-, c, b) Cl^-, e, f) Br^-, g, h) I^-, i, j) NO_3^--loaded and k, l) salt-free PNIPMAM thin films upon a, c, e, g, I, k) D_2O swelling (stage II) and b, d, f, h, j, l) subsequent vapor switching (stage VI). The characteristic bands listed in Table S3 are indicated by blue lower-case letters in c) and d), and the black arrows highlight the change of corresponding characterized signals.

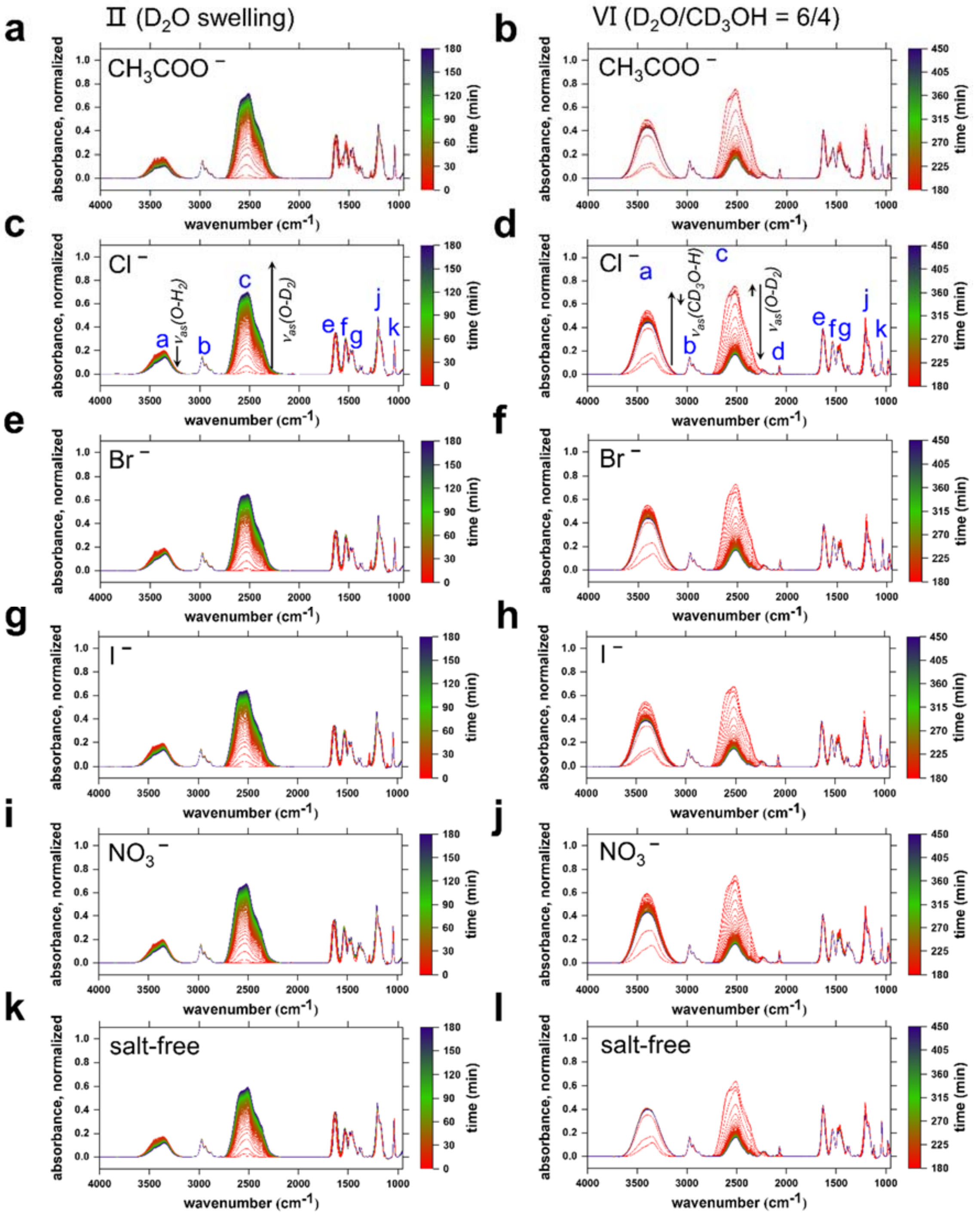

Figure S6. FT-IR spectra of a, b) CH_3COO^-, c, b) Cl^-, e, f) Br^-, g, h) I^-, i, j) NO_3^--loaded and k, l) salt-free PSBP-*b*-PNIPMAM thin films upon a, c, e, g, I, k) D_2O swelling (stage II) and b, d, f, h, j, l) subsequent vapor switching (stage VI). The characteristic bands listed in Table S3 are indicated by blue lower-case letters in c) and d), and the black arrows highlight the change of corresponding characterized signals.

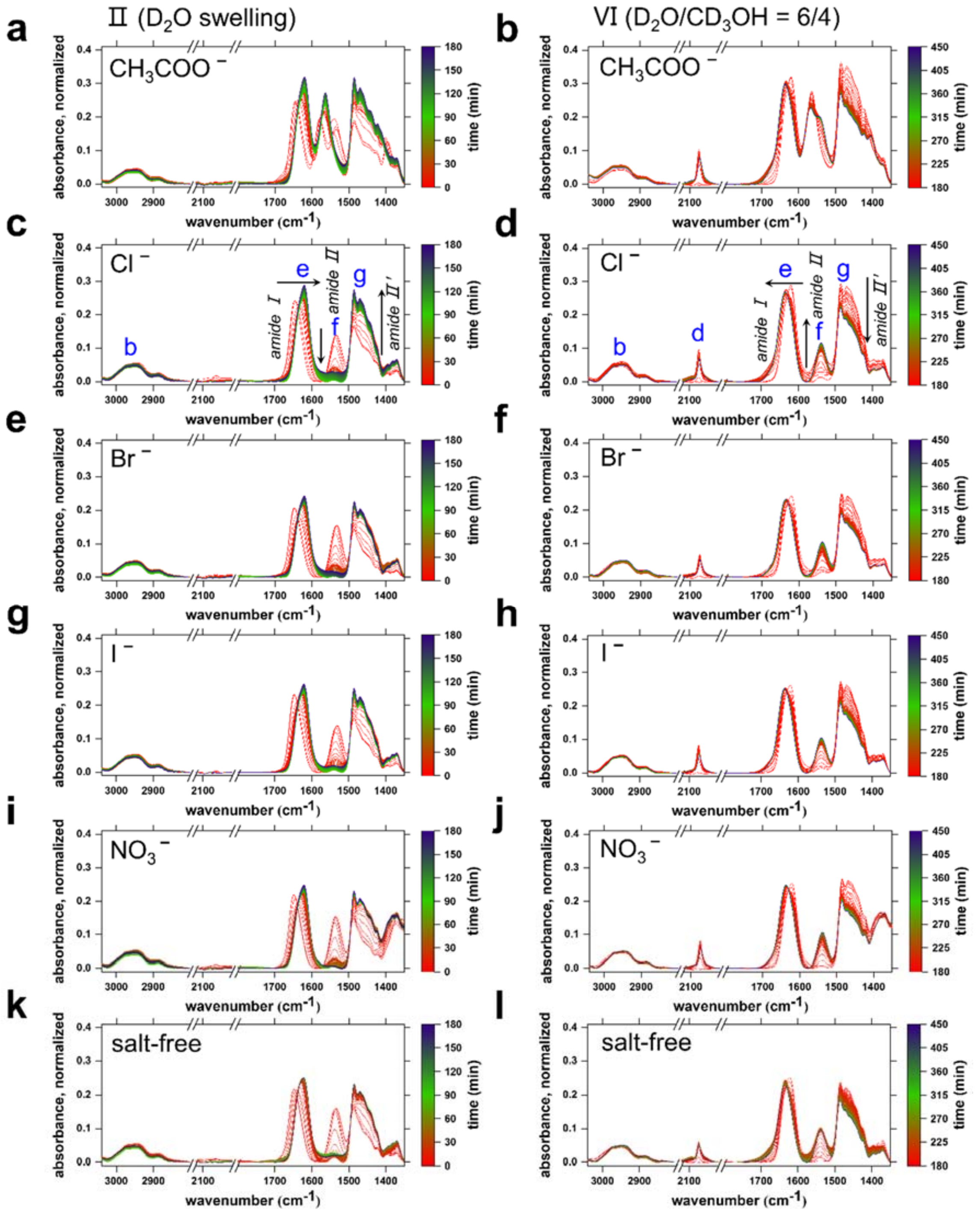

Figure S7. Enlarged FT-IR spectra of a, b) CH_3COO^-, c, b) Cl^-, e, f) Br^-, g, h) I^-, i, j) NO_3^--loaded and k, l) salt-free PSBP thin films upon a, c, e, g, I, k) D_2O swelling (stage II) and b, d, f, h, j, l) subsequent vapor switching (stage VI). The black arrows in c) and d) highlight the change of corresponding amide groups.

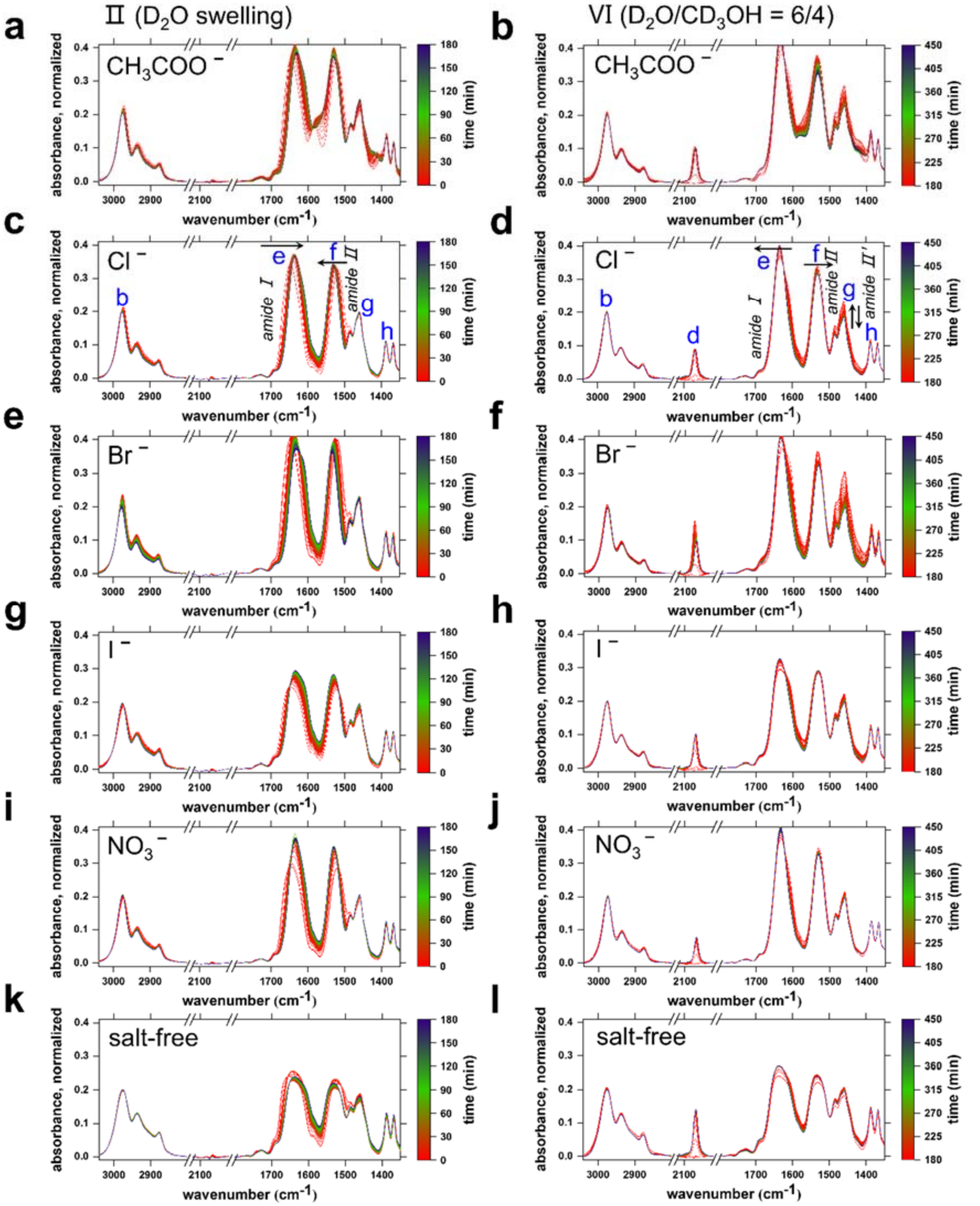

Figure S8. Enlarged FT-IR spectra of a, b) CH_3COO^-, c, b) Cl^-, e, f) Br^-, g, h) I^-, i, j) NO_3^--loaded and k, l) salt-free PNIPMAM thin films upon a, c, e, g, I, k) D_2O swelling (stage II) and b, d, f, h, j, l) subsequent vapor switching (stage VI). The black arrows in c) and d) highlight the change of corresponding amide groups.

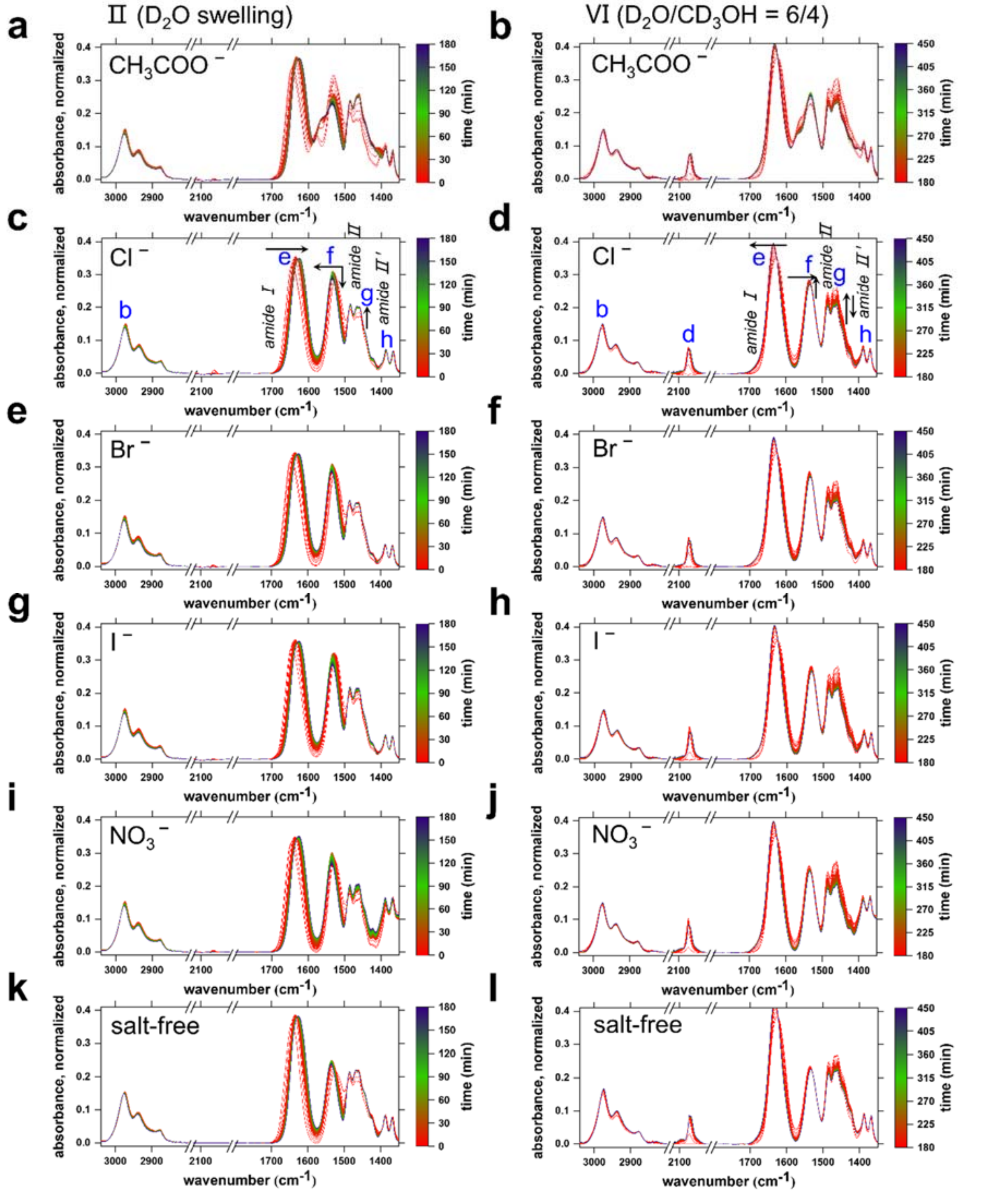

Figure S9. Enlarged FT-IR spectra of a, b) CH_3COO^-, c, b) Cl^-, e, f) Br^-, g, h) I^-, i, j) NO_3^--loaded and k, l) salt-free PSBP-*b*-PNIPMAM thin films upon a, c, e, g, I, k) D_2O swelling (stage II) and b, d, f, h, j, l) subsequent vapor switching (stage VI). The black arrows in c) and d) highlight the change of corresponding amide groups.

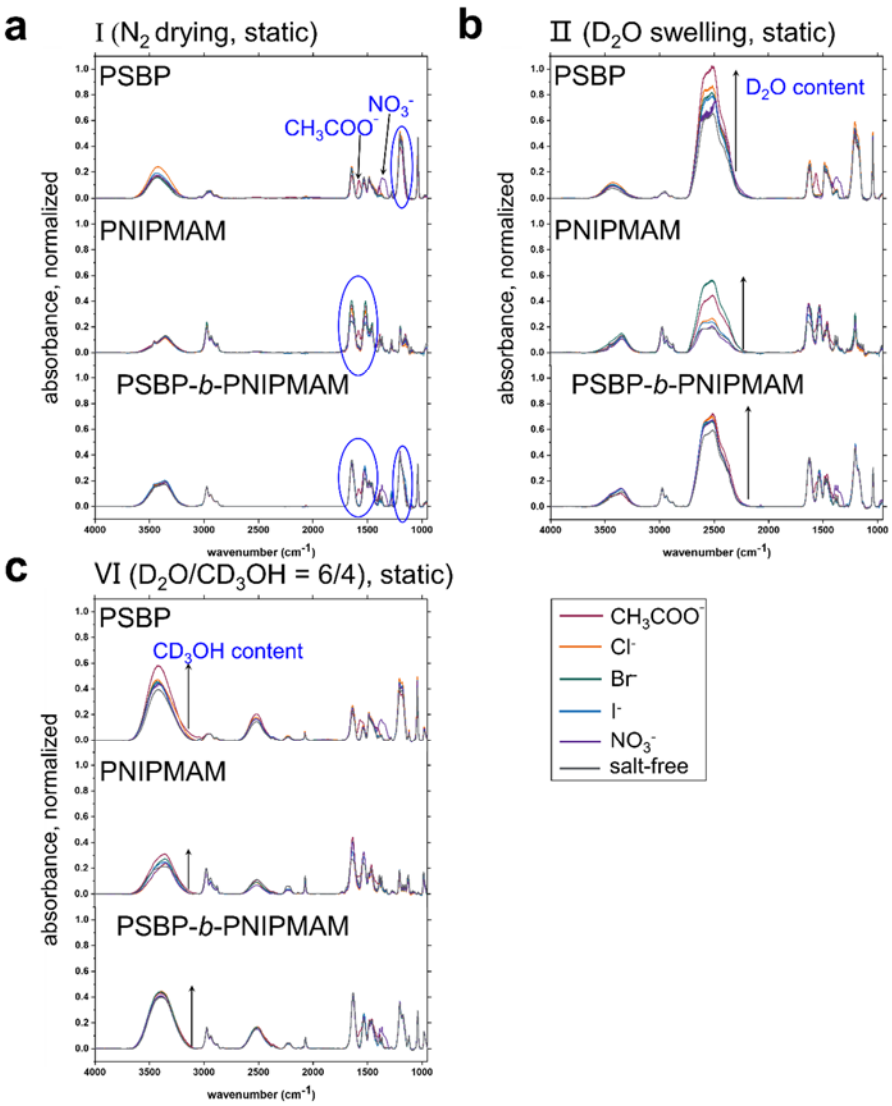

Figure S10. Static FT-IR spectra of salt-loaded and salt-free PSBP, PNIPMAM and PSBP-*b*-PNIPMAM thin film upon a) N_2 drying (stage I), b) D_2O vapor swelling (stage II) and c) vapor switching (stage VI). The static FT-IR spectra at stage I are recorded as the first spectrum during the D_2O swelling process, and the static FT-IR spectra at stage II and VI are recorded as the last spectrum during the D_2O swelling process and vapor switching process respectively. The spectra of the CH_3COO^-, Cl^-, Br^-, I^-, NO_3^--loaded and salt-free samples are colored in red, orange, green, blue, purple, and grey respectively.

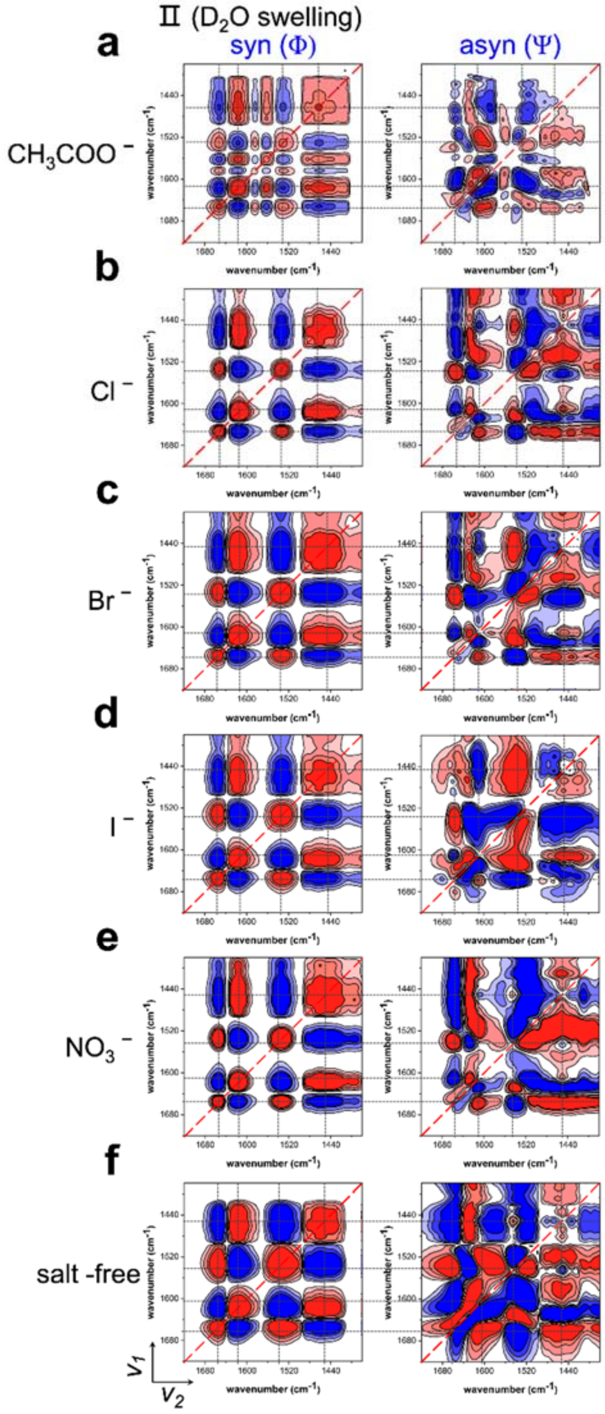

Figure S11. 2D FT-IR correlation contour maps of a) CH_3COO^-, b) Cl^-, c) Br^-, d) I^-, e) NO_3^--loaded and f) salt-free PSBP thin films upon D_2O swelling process (stage II) in the region of 1720 – 1380 cm^{-1}.

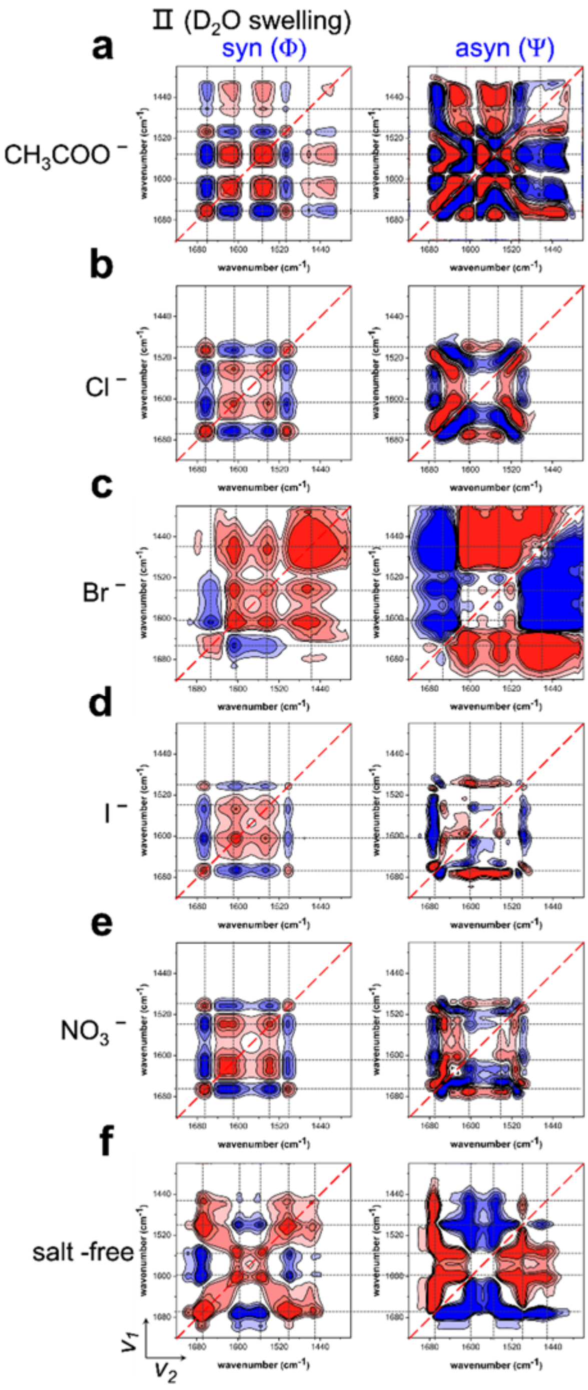

Figure S12. 2D FT-IR correlation contour maps of a) CH_3COO^-, b) Cl^-, c) Br^-, d) I^-, e) NO_3^--loaded and f) salt-free PNIPMAM thin films upon D_2O swelling process (stage II) in the region of 1720 – 1380 cm^{-1}.

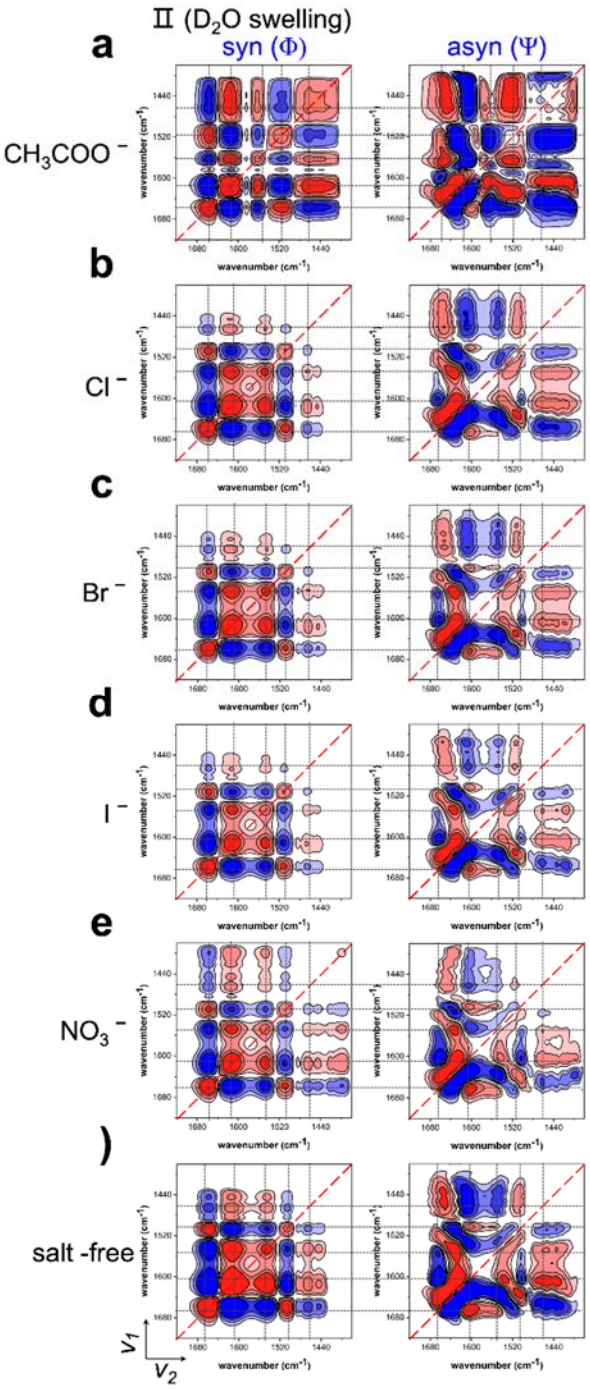

Figure S13. 2D FT-IR correlation contour maps a) CH_3COO^-, b) Cl^-, c) Br^-, d) I^-, e) NO_3^--loaded and f) salt-free PSBP-*b*-PNIPMAM thin films upon D_2O swelling process (stage II) in the region of 1720 – 1380 cm^{-1}.

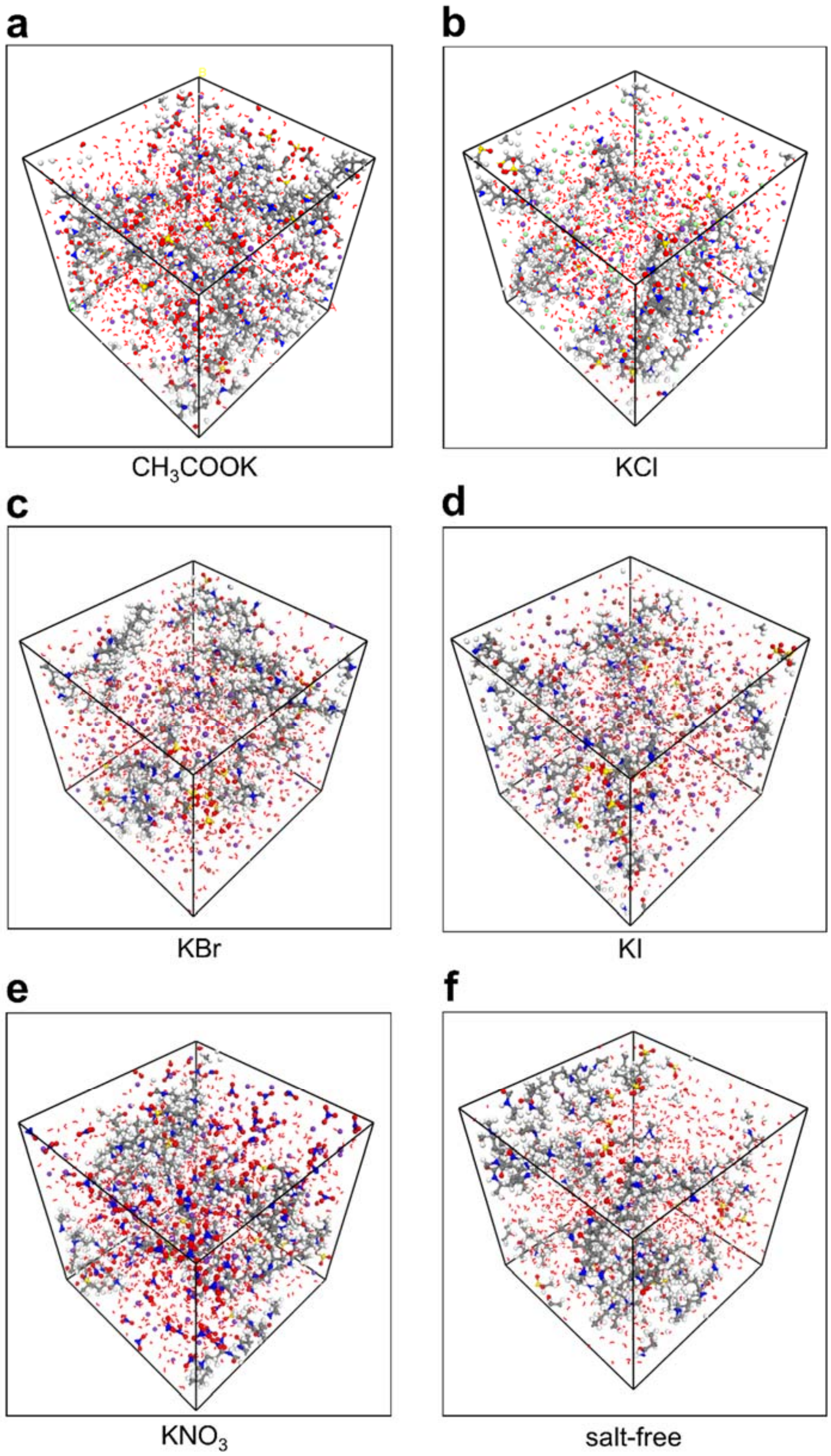

Figure S14. Snapshots of the used simulation boxes for a) CH_3COO^-, b) Cl^-, c) Br^-, d) I^-, e) NO_3^--loaded and f) salt-free PSBP-*b*-PNIPMAM chains.

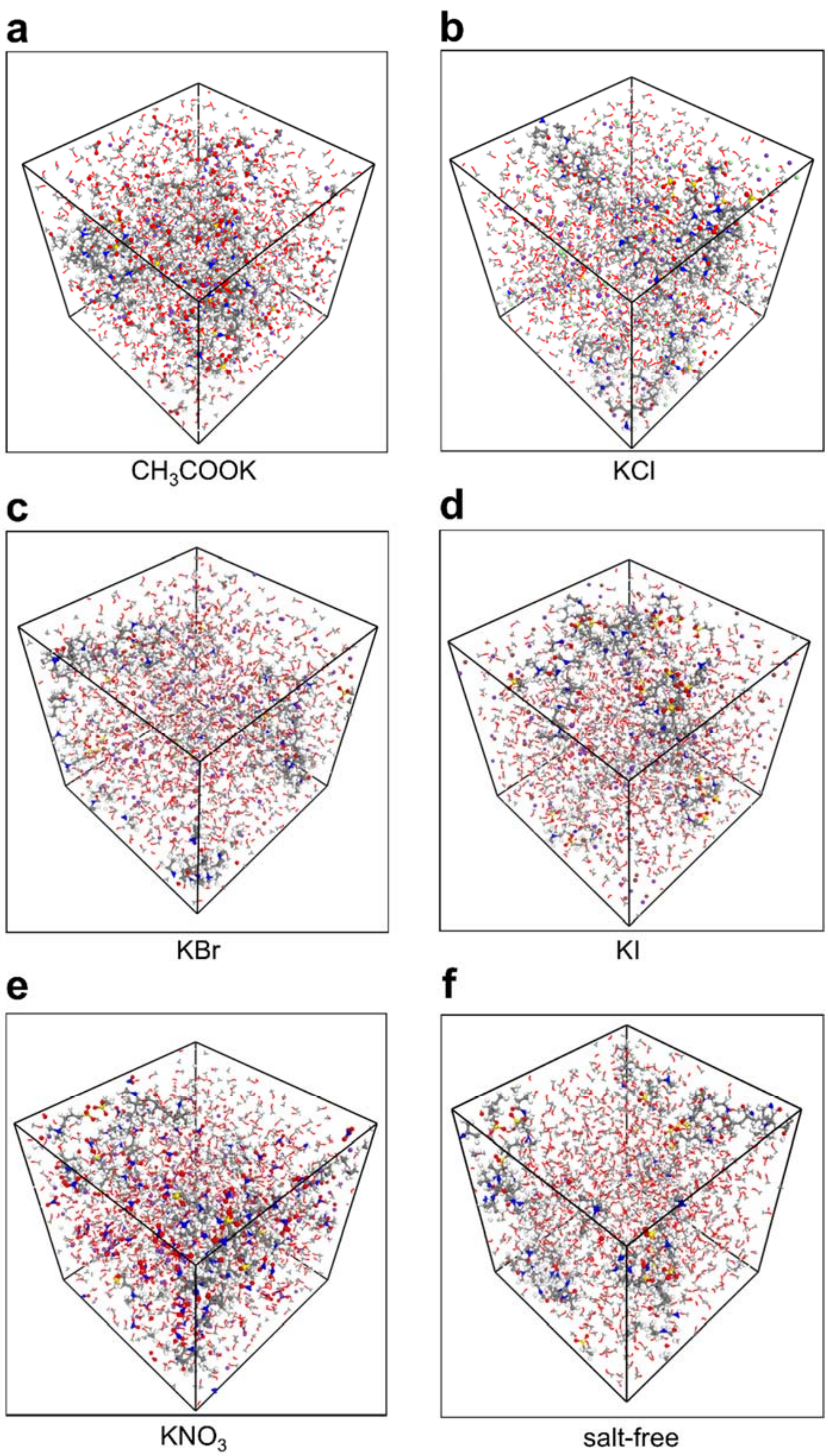

Figure S15. Snapshots of the used simulation boxes for a) CH_3COO^-, b) Cl^-, c) Br^-, d) I^-, e) NO_3^--loaded and f) salt-free PSBP-*b*-PNIPMAM chains.

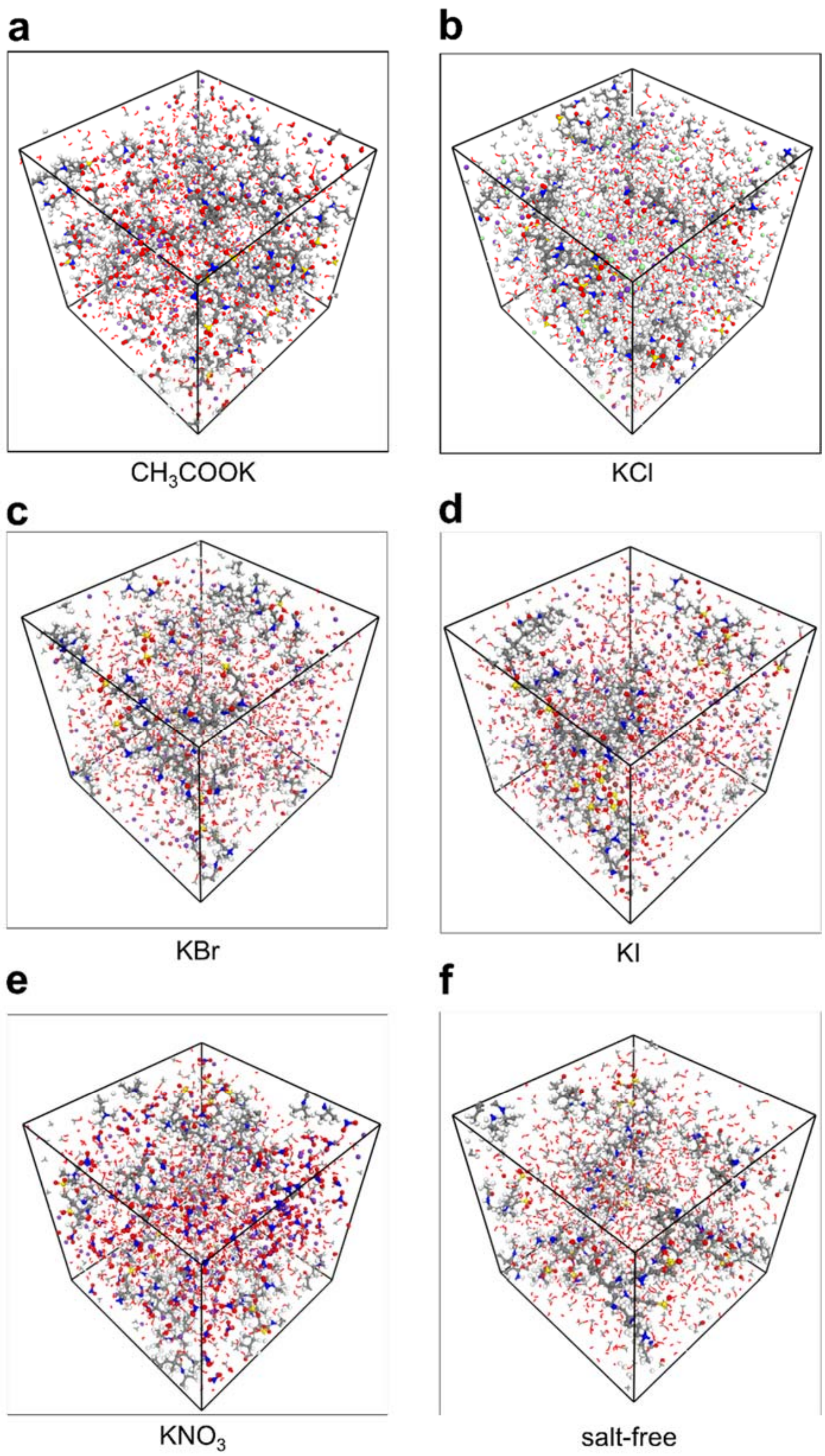

Figure S16. Snapshots of the used simulation boxes for a) CH_3COO^-, b) Cl^-, c) Br^-, d) I^-, e) NO_3^--loaded and f) salt-free PSBP-*b*-PNIPMAM chains.

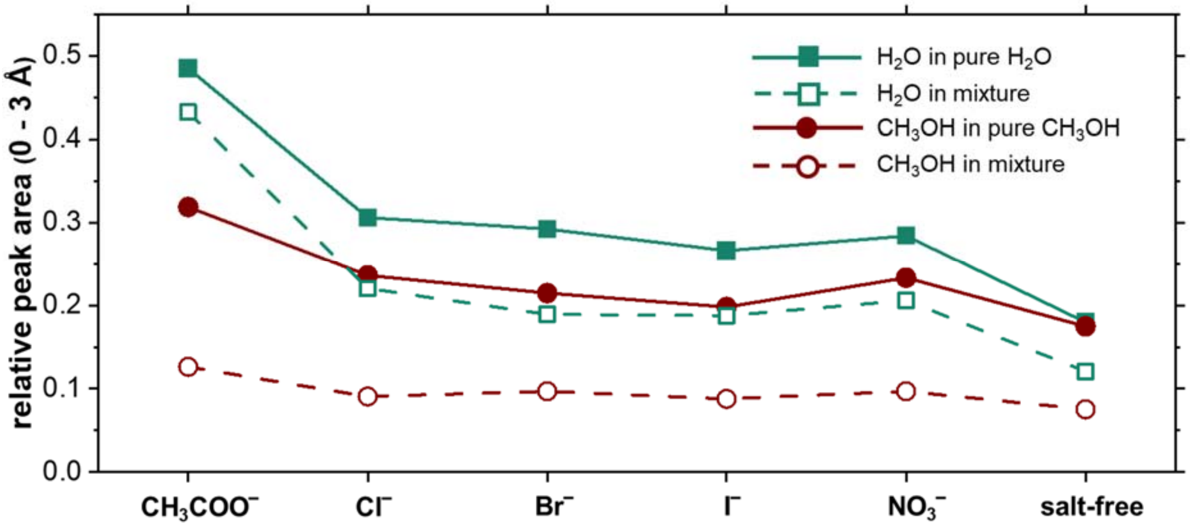

Figure S17. The relative accessibility is plotted as green squares of H_2O shells and red circles for CH_3OH shells, connected by solid lines in pure atmosphere and by dash lines in mixed atmosphere respectively.

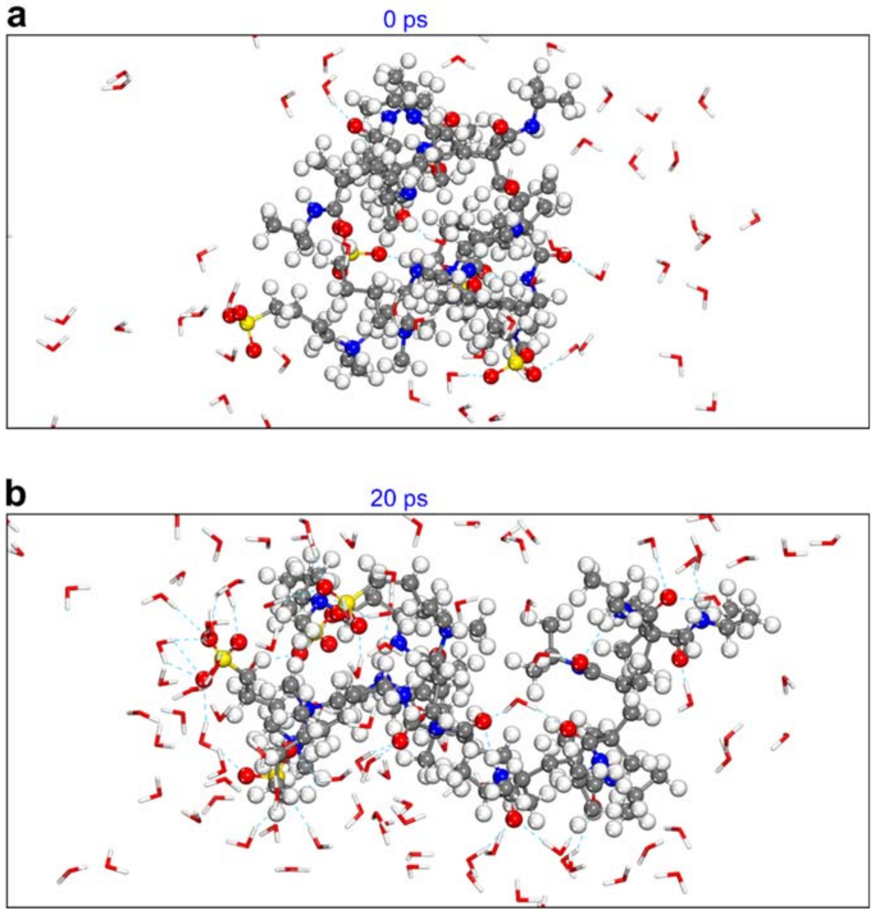

Figure S18. Snapshots illustrating the conformations of salt-free PSBP-*b*-PNIPMAM chains at a) 0 and b) 20 ps. The atoms of N, O and S are colored dark blue, red and yellow respectively. The dashed lines in light blue represent the formed hydrogen bonds.

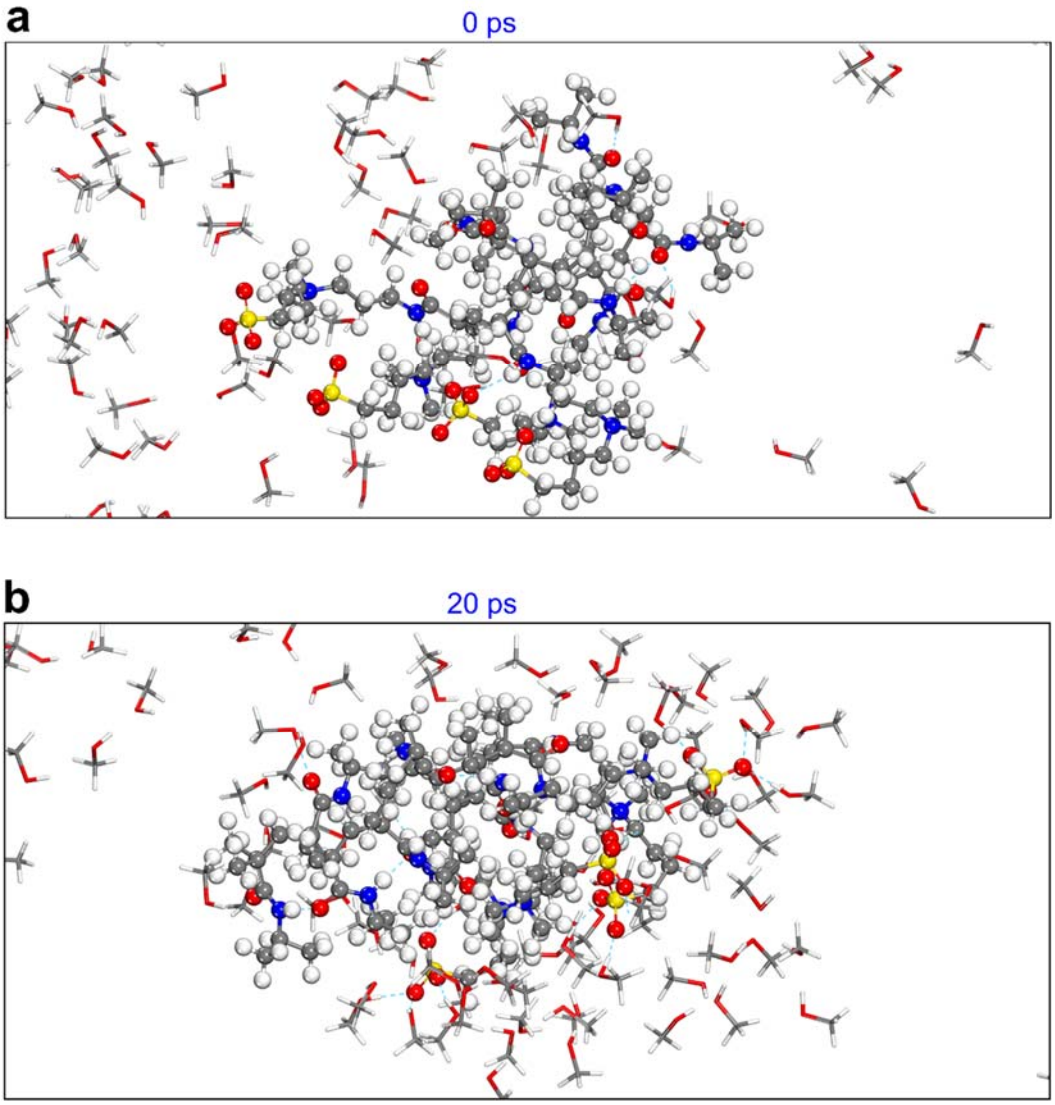

Figure S19. Snapshots illustrating the conformations of salt-free PSBP-*b*-PNIPMAM chainsat a) 0 and b) 20 ps. The atoms of N, O and S are colored dark blue, red and yellow respectively. The dashed lines in light blue represent the formed hydrogen bonds.

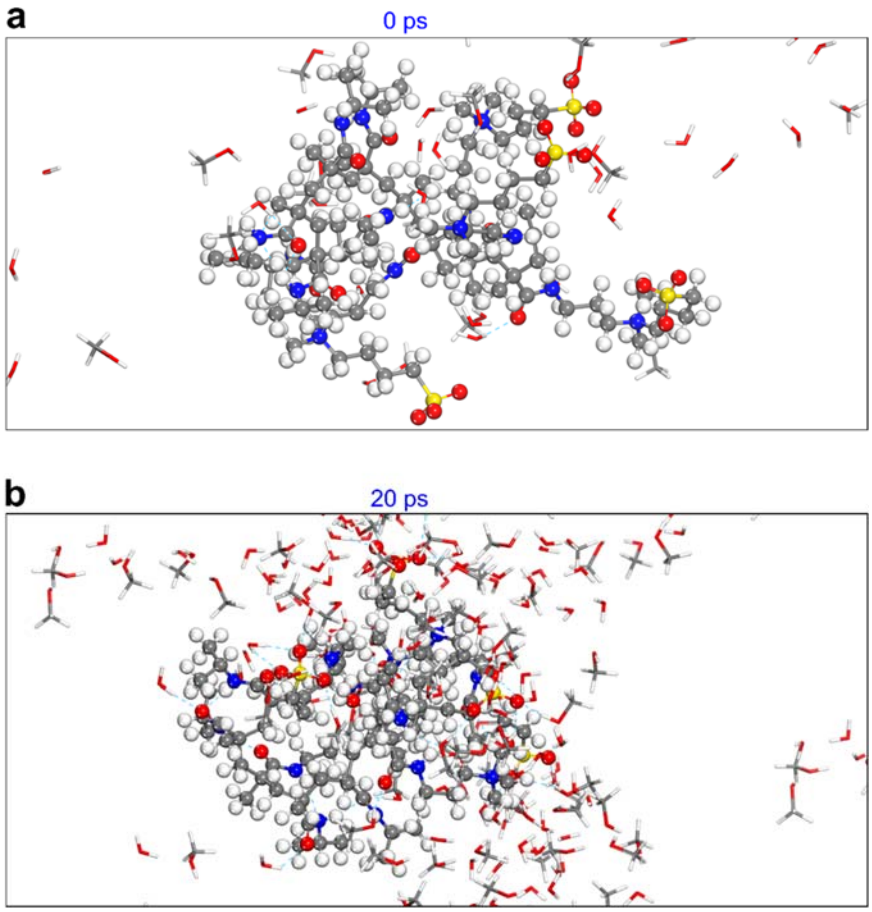

Figure S20. Snapshots illustrating the conformations of salt-free PSBP-*b*-PNIPMAM chains at a) 0 and b) 20 ps. The atoms of N, O and S are colored dark blue, red and yellow respectively. The dashed lines in light blue represent the formed hydrogen bonds.

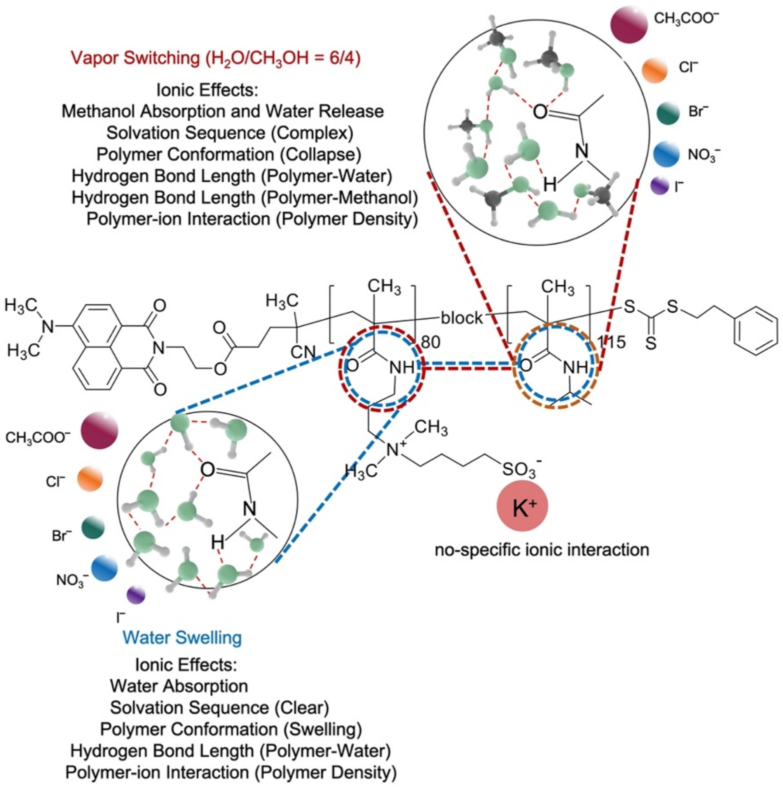

Figure S21. Schematic diagram summarizing the proposed mechanisms of polymer solvation behavior in the presence of salt.

Supplementary References

[1] N. S. Vishnevetskaya, V. Hildebrand, M. A. Dyakonova, B.-J. Niebuur, K. Kyriakos, K. N. Raftopoulos, Z. Di, P. Müller-Buschbaum, A. Laschewsky, C. M. Papadakis, *Macromolecules* **2018**, *51*, 2604-2614.

[2] a) T. Widmann, L. P. Kreuzer, G. Mangiapia, M. Haese, H. Frielinghaus, P. Müller-Buschbaum, *Rev. Sci. Instrum.* **2020**, *91*, 113903; b) P. Wang, C. Geiger, J. Reitenbach, A. Vagias, L. P. Kreuzer, S. Liang, R. Cubitt, V. Hildebrand, A. Laschewsky, C. M. Papadakis, P. Müller-Buschbaum, *Macromolecules* **2023**, *56*, 4087-4099.

[3] P. Wang, C. Geiger, L. P. Kreuzer, T. Widmann, J. Reitenbach, S. Liang, R. Cubitt, C. Henschel, A. Laschewsky, C. M. Papadakis, P. Müller-Buschbaum, *Langmuir* **2022**, *38*, 6934-6948.

[4] A. Nelson, *J. Appl. Crystallogr.* **2006**, *39*, 273-276.

[5] C. Geiger, J. Reitenbach, L. P. Kreuzer, T. Widmann, P. Wang, R. Cubitt, C. Henschel, A. Laschewsky, C. M. Papadakis, P. Müller-Buschbaum, *Macromolecules* **2021**.

[6] M. Reufer, P. Díaz-Leyva, I. Lynch, F. Scheffold, *Eur Phys J E Soft Matter* **2009**, *28*, 165-171.

[7] a) I. Noda, *Journal of the American Chemical Society* **1989**, *111*, 8116-8118; b) I. Noda, *Chin. Chem. Lett.* **2015**, *26*, 167-172; c) I. Noda, A. E. Dowrey, C. Marcott, G. M. Story, Y. Ozaki, *Appl. Spectrosc.* **2000**, *54*, 236A-248A.

[8] a) T. Zheng, J. Xiong, X. Shi, B. Zhu, Y.-J. Cheng, H. Zhao, Y. Xia, *Energy Storage*

Mater. **2021**, *38*, 599-608; b) Z. Wang, Y. Sun, Y. Mao, F. Zhang, L. Zheng, D. Fu, Y. Shen, J. Hu, H. Dong, J. Xu, *Energy Storage Mater.* **2020**, *30*, 228-237.
